# Trans,trans-farnesol, an antimicrobial natural compound, improves glass ionomer cement properties

**DOI:** 10.1371/journal.pone.0220718

**Published:** 2019-08-20

**Authors:** Aline Rogéria Freire de Castilho, Pedro Luiz Rosalen, Isaac Jordão de Souza Araújo, Igor Lebedenco Kitagawa, Cecilia Atem Gonçalves de Araújo Costa, Malvin N. Janal, Marcelo Corrêa Alves, Simone Duarte, Paulo Noronha Lisboa Filho, Rafael Nobrega Stipp, Regina Maria Puppin-Rontani

**Affiliations:** 1 Department of Pediatric Dentistry, Piracicaba Dental School, University of Campinas, Piracicaba, São Paulo, Brazil; 2 Department of Physiological Sciences, Piracicaba Dental School, University of Campinas, Piracicaba, São Paulo, Brazil; 3 Department of Restorative Dentistry, Piracicaba Dental School, University of Campinas, Piracicaba, São Paulo, Brazil; 4 Department of Physics, School of Science, State University of São Paulo, Bauru, São Paulo, Brazil; 5 Department of Restorative Dentistry, Federal University of Ceará, Fortaleza, Ceará, Brazil; 6 Department of Epidemiology and Health Promotion, New York University College of Dentistry, New York, New York, United States of America; 7 Superior School of Agriculture "Luiz de Queiroz" Technical Section of Information Technology, University of São Paulo, Piracicaba, São Paulo, Brazil; 8 Department of Cariology, Operative Dentistry and Dental Public Health, Indiana University, Purdue University Indianapolis, School of Dentistry, Indianapolis, Indiana, United States of America; 9 Department of Oral Diagnosis, Piracicaba Dental School, State University of Campinas, Piracicaba, São Paulo, Brazil; University of Bern, SWITZERLAND

## Abstract

A series of experiments were conducted to characterize a novel restorative material. We explored the effect on biological, physical and chemical properties of glass ionomer cement (GIC) adding-the naturally occurring *tt*-farnesol (900 mM). Two groups were accomplished for all assays: GIC+*tt*-farnesol and GIC (control). Biological assays: 1) agar diffusion against some cariogenic bacteria; 2) *S*. *mutans* biofilm formation and confocal laser scanning microscopy-CLSM. 3) *gtfB*, *gtfC*, *gtfD*, *gbpB*, *vicR*, *and covR* expression; 4) MTT and microscopic morphology. Physical properties assays: 1) roughness; 2) hardness; 3) compressive strength and 4) diametral tensile strength. Chemical assay: Raman spectroscopy. The adding of *tt*-farnesol to GIC led to larger zones of inhibition (p<0.05), biofilms with a short-term reduction in bacterial viability but similar biomass (p>0.05). Polysaccharides levels increased over time, similarly over groups (p>0.05). Viable and non-viable *S*. *mutans* were seen on the specimens’ surface by CLSM but their virulence was not modulated by *tt*-farnesol. The *tt*-farnesol increased the HaCaT cell viability without impact on compressive and diametral tensile strength and roughness although the hardness was positively affected (p<0.05). Raman confirmed the presence of *tt*-farnesol. The incorporation of *tt*-farnesol into GIC inhibited the growth of cariogenic bacteria but had a little effect on the composition, structure and physiology of the biofilm matrices. Also, the *tt*-farnesol increased the hardness and the biocompatibility of the GIC, not influencing negatively other physical properties of the restorative material.

## Introduction

Glass ionomer cement (GIC) is one of the dental restorative materials options for replacing tooth tissue loss from caries lesion [1]. Clinically, the GIC exhibits acceptable mechanical, physical and biological properties as filling material, and also releases fluoride in oral cavity [1]. However, that fluoride release is efficient only for controlling the demineralization on the adjacent tooth surfaces, since their action on biofilm is still limited [2]. Improvement of antimicrobial properties without jeopardize the physical properties of the GIC’s is important as dental treatment must correlate the cost and the functionality of the material, the range of use as well as the possibility of indicating the material for public health services and people lack access to dental care [3].

Nowadays, dental researches have been focused on the discovery of novel natural therapies with pharmacological and biological activities [4], including anti-cariogenic effect [5]. In this context, natural products and their derivatives are the most successful strategy for the development of therapeutic agents. They have been widely used in pharmaceutical industries in the search for new secure and effective medicinal approaches [4]. Therefore, the combining of natural compounds with existing formulations may enhance the antimicrobial action of the dental material, effectively preventing and controlling the caries lesion and biofilm formation on the material surface. Furthermore, it is important to highlight that the incorporation of natural compounds to fluoride highly increase the inhibition of cariogenic microorganisms but without suppressing of local microbiota [6].

Although a range of microorganisms are involved in carious process [7], *Streptococcus mutans* is still considered the main contributor to the initiation of the lesion, providing the matrix of biofilm [8]. Basically, *S*. *mutans* is involved in a dynamic process mediated by sucrose [8] that includes the instability of molecular interaction of the tooth surface and microbial biofilm, resulting in hard tissue loss [9]. Overall, biofilms are three-dimensional structures made of a combination of microorganisms adhered to each other, to a surface and embedded within an extracellular polymeric substance (EPS) [10]. It is known that the production of the EPS increases in the presence of sucrose and glucosyltransferase (GTF) activity, building a physical barrier to macrophages, phagocytes as well as antimicrobial substances [9]. Thus, if the biofilm had been disorganized, the mineral loss on tooth surface would stop and the carious lesion would be arrested [11]. Theoretically, the use of formulations containing potent inhibitors of GTFs may also inhibit the polysaccharide production in dental biofilms [12].

Trans,trans-farnesol (*tt*-farnesol, 3,7,11-trimethyl-2,6,10-dodecatrien-1-ol) is a bioactive sesquiterpene alcohol naturally occurring in pine (Pinus), chamomile (*Matricaria chamomilla* L., Asteraceae), arnica (Arnica montana, Asteraceae) artemisia (Artemisia annua, Asteraceae), a bee resin called propolis, and citrus fruits [13]. Their use is mostly exploited in cosmetics and non-cosmetic products [13]. The potential anti-caries efficacy of that terpenoid is related to the inhibition of acid production and glucan synthesis by *S*. *mutans* [14] since *tt*-farnesol may increase the proton permeability of streptococcal membranes, thus, promoting the decrease of GTFs secretion [15].

Given the relevance of caries control, the aim of this study was to determine how the characterization of a GIC containing a natural product affects several material properties, attempting to elucidate their antimicrobial effect, cytotoxicity and physical performance. When adding the natural occurring product to the GIC we hypothesized that: (1) *tt*-farnesol would increase the antimicrobial activity of the GIC; (2) *tt*-farnesol would modify the composition, structure and physiology of *S*. *mutans* biofilm; (3) the experimental GIC is biologically compatible with human keratinocytes and; (4) the physical and chemical properties of the GIC would not be negatively modified by *tt*-farnesol incorporation.

## Materials and methods

For this study, a conventional glass ionomer cement (GIC; Fuji IX GP, GC America, USA; Batch #1212051) was modified by adding 900 mM sesquiterpene *tt*-farnesol (Sigma–Aldrich, Steinheim, Germany) to the liquid of the GIC while keeping original powder/liquid ratio (3.6 g: 1.0 g). Then, the powder and the liquid containing *tt*-farnesol were hand-mixed for 30 s using a plastic spatula and non-absorbent paper according to the manufacturer instructions. All procedures were carried out in a biosafety cabinet (Telstar, Terrassa, Spain). The control group was GIC with no natural product incorporation [16–18].

### Specimen preparation

Standard round-shaped specimens (5 mm x 2 mm) containing or not *tt*-farnesol were prepared by using silicon molds, at a temperature of 23 °C ± 1 °C, and a relative humidity of 50% ± 5%. After the setting reactions were completed, the specimens were polished using Sof-Lex discs (3M ESPE, St Paul, MN, USA), washed through sonication for 10 min, 1 min soaked in 70% ethanol for disinfection, dried and exposed to the UV radiation for physical sterilization before antimicrobial, cytotoxicity and physical tests.

### Antimicrobial assays

#### Agar diffusion test

*Streptococcus mutans* UA159, *Lactobacillus acidophilus* (ATCC IAL523), *Lactobacillus casei* (ATCC 193) and *Actinomyces naeslundii* (ATCC 12104) were cultured from frozen stock on brain–heart infusion broth (BHI; DIFCO Laboratories, Detroit, MI, USA) for 24 h at 37 °C in 5% CO_2_. After confirming the viability and the absence of contamination by plating in specific media and Gram techniques, cultures were again grown in BHI for 18–24 h at 37 °C and adjusted to a concentration of 1 x 10^8^ cells/mL. Petri dishes containing a base layer of BHI agar mixed with each inoculum were prepared. Following, wells measuring 5 mm in diameter were made in each plate and completely filled with the GIC + *tt*-farnesol (experimental group) or GIC (control). Then, the 0.2% chlorhexidine digluconate was applied on sterile filter paper discs as a control of the experiment. After the period of incubation (24 h, at 37 °C), inhibition zones around the materials were measured using a digital caliper [16,17]. Agar diffusion test was performed in triplicate on at least three independent experiments.

#### Biofilm model and morphology analysis

*S*. *mutans* biofilm model was based on previous methodology [19] with some modifications. Briefly, after being coated with sterile salivary pellicle, two samples of each experimental (GIC + *tt*-farnesol) and control group (GIC) were incubated with *S*. *mutans* UA 159 in BHI broth supplemented with 10% sucrose at 37 °C for 1, 3 and 5 days in a 5% supplemented CO_2_ environment. The medium pH was measured daily. Then, biofilm bacterial viability (colony forming unities-CFU/mg of biofilm), biomass (dry weight-DW), and biochemical composition (insoluble-ASP and water-soluble-WSP extracellular polysaccharides) was analyzed by colorimetric methods. Biofilm model was performed in duplicate on at least four independent experiments.

The morphology of live and dead bacteria on biofilm surface was analyzed by confocal laser scanning microscopy (CLSM; Leica TCS SP1, Leica Lasertechnik GmbH, Heidelberg, Germany) using HCX APOL U-V-I 40X/0.8-numerical-aperture water immersion objective. Biofilms were stained with a live/dead BacLight bacterial viability kit (Molecular Probes. Invitrogen, Eugene, Oregon. USA) in accordance with the manufacturer. Afterwards, the samples were incubated at room temperature in the dark for 15 min and examined under a CLSM [19].

#### Gene expression (RT-qPCR)

Based on previous study [20], sixty-six specimens of GIC containing *tt*-farnesol were made for the gene expression analysis. Following the biofilm model described before, after 24 h of biofilm growth in BHI supplemented with 10% sucrose, the specimens were removed from culture plates and transferred to tubes with 2 ml of 0.9% NaCl. Tubes were then vortexed (2.800 rpm/10 s) in order to detach the cells. Following, the supernatant was transferred to 2 ml micro-tubes and were centrifuged (2 min, 4 °C, ≈16000 g) for cell pellet precipitation. The saline solution was then discarded and cell pellet was immediately stored at -80 °C until RNA purification. Cell pellet (n = 6) was obtained from biofilms formed over three specimens per group.

Subsequently, the RNA total purification was performed using ≈ 0.16 g of 0.1 mm diameter zirconia beads (Biospec, Bartlesville, USA), combined with 220 μL TE buffer on a Mini-bead beater apparatus (Biospec). RNeasy Mini Kit protocol (Qiagen, Hilden, Germany) was used for total RNA purification. The RNA was then converted to cDNA using iScript Reverse Transcriptase (Bio-Rad Laboratories, Hércules, USA). Reverse transcriptase reactions were prepared according to manufacture recommendation using 60 ng of RNA from the sample and were incubated at 25 °C for 5 min, 42 °C for 30 min and 85 °C for 5 min. Furthermore, an additional reaction was prepared in the absence of iScript to assess the absence of genomic DNA contamination of each sample. The converted cDNA was stored at -20 °C for further analysis of gene expression.

The RT-qPCR reactions were performed using specific primers for the following genes: *gtfB*, *gtfC*, *gtfD*, *gbpB*, *vicR*, and *covR* [21] in a StepOne Real-Time PCR Systems (Applied Biosystems, UK). From each sample, 1 μL of cDNA was placed in a 48-plate well with 9 μL of a solution containing 3.4 μL water free RNase and DNase, 0.6 μL of primer and 5 μL of SYBR Green PCR Master Mix (Applied Biosystems). Standard curves (300, 30, 3, 0.3, and 0.03) were performed for each primer pair assay. All analyzed genes were normalized and their expression was calculated using the 16s RNA as the reference gene.

#### Cell incubation and cytotoxicity of the materials

HaCaT (Human adult low Calcium high Temperature Keratinocytes) cells were routinely cultured in Dulbecco’s Modified Eagle’s Medium (DMEM; Sigma Chemical Co., St. Louis, MO, USA) supplemented with 10% fetal bovine serum (FBS; Gibco, Grand Island, NY, USA), with 100 IU/mL penicillin and 100 mg/mL streptomycin (Vitrocell Embriolife, Campinas, SP, Brazil) in humidified atmosphere containing 5% CO_2_. Cells were seeded at a cell density of 5 x 10^4^ cells/well and cell viability and proliferation were evaluated by MTT test.

For the MTT test, the cells were seeded on 24-well plate in DMEM with 10% FBS before exposure to specimens (experimental and control groups). Twenty-four hours later, 0.3 mg/mL MTT was added in each well, and cells were incubated for 3 h at 37 °C. Absorbance was measured with a micro spectrophotometer (ASYS UVM340, Biochrom Ltd., Cambridge, UK) at 570 nm. The MTT assay was performed in duplicate on three independent experiments [22].

Images of the live and dead cells (Live/Dead, Molecular Probes Life Technologies, USA) were carried out by means of a fluorescence microscope (Zeiss Axiovert 40 CFL, Germany) coupled to an ECM AxioCam camera (Carl Zeiss, Germany). Green labeled cells (live) were visualized using a filter in a wavelength range of 450–490 nm (Ex)/ 515–565 nm (Em). Red labeled cells (dead) were visualized in 528–546 nm (Ex) / 590–617 nm (Em) wavelength range. For each experimental condition, three images were taken at random areas of the slide in triplicate.

#### Physical properties

Specimens (n = 10/group/test) were subjected to four different physical assays. The roughness (Ra-μm) of the specimen’s surface was quantified using a roughness-measuring instrument (Surfcorder SE1700, Kosaka Corp, Tokyo, Japan). A needle moved at a constant speed of 0.5 mm/sec with a load of 0.7 mN and a cut-off value at 0.25 mm. Hardness tests were carried out with a hardness tester (Shimatzu 2000, Tokyo, Japan), with a 25-g load applied by the a Knoop indenter for 15 s. Three readings (KHN) were taken on the top surface of the material at 100 μm distance from each other.

Instron universal test machine (4411, Instron Co., Canton, Mass, USA) was used for assessment of compressive strength and diametral tensile strength tests. Specimens were submitted to testing machine and the parameters for measurement of the assays were set at a crosshead speed of 1.0 mm/min for the compressive strength and at 0.5 mm/min crosshead speed for the diametral tensile strength. To convert the data recorded in kgf/cm^2^ into MPa, values of compressive strength were calculated by dividing the load of failure over the cross-sectional area, while diametral tensile strength was calculated using the equation: (2 × Load)/(3.14 × Diameter × Thickness) [17,18, 22].

#### Raman spectroscopy

For confirmation of the *tt*-farnesol incorporation, the spectra of the specimens were conducted using a Renishaw micro-Raman system model inVia equipped with Leica microscope which allows the acquisition of a spectrum in a point with spatial resolution of about 1 *μm*^2^ using a 50 x objective lens and CCD detector with a collection time of 10 s. The laser line at 514.5 nm was used with 1800 lines/mm grating resulting in a spectral resolution of about 4 cm^-1^ [23].

#### Statistical analysis

The effect of GIC containing *tt*-farnesol on mean measures of the *S*. *mutans* biofilm viability and weight, and also measures of ASP and WSP were compared between groups (n = 4 per group) after 1, 3 and 5 days of formation and for MTT using a 2-way mixed model ANOVA. Homogeneity of variances was confirmed prior to statistical analysis (Levene’s test). Group differences on the agar diffusion test, physical properties and gene expression were evaluated with 1-way-ANOVA. When appropriate, statistical relations were observed using Shapiro-Wilk, Tukey and Tukey-Kramer post-hoc analyses. The level of significance was set at 5% in all tests (SAS Institute Inc. The SAS System, release 9.4, SAS Institute Inc., Cary:NC, 2012)(S1–S13 Texts).

## Results

### Antimicrobial assays

#### Agar diffusion test

The mean values and standard deviation of the inhibition zones for experimental and control groups for each microbe are shown in Table 1. GIC showed no zone of inhibition for the growth of *L*. *acidophilus*, *L*. *casei and A*. *naeslundii*. The *tt*-farnesol group exhibited the larger zones of inhibition than GIC or chlorhexidine, indicating antimicrobial activity against *S*. *mutans*, *L*. *acidophilus*, *L*. *casei and A*. *naeslundii* (p < 0.05).

**Table 1:** Antibacterial activity of GIC containing or not *tt*-farnesol on cariogenic bacteria (mean ± standard deviation).

Groups	Microorganism/ Inhibition zone (inch)			
	*S*. *mutans* UA 159	*L*. *acidophilus* ATCC IAL523	*L*. *casei* ATCC 193	*A*. *naeslundii* ATCC 12104
0.2% Chlorexidine Digluconate (3.8 mM)	4.3±0.1^a^*	4.2±0.8^a^	2.0±0.5^a^	3.7±0.0^a^
GIC*+ tt-*farnesol (900 mM)	14.8±1.5^b^	8.14±1.0^b^	1.03±0.1^b^	3.4±0.2^b^
GIC	0.3±0.0^c^	0^c^	0^c^	0^c^

* For each microorganism, different lower-case letters indicate mean differences between treatments (p < 0.05).

### Biofilm model and morphology analysis

**Fig 1:**
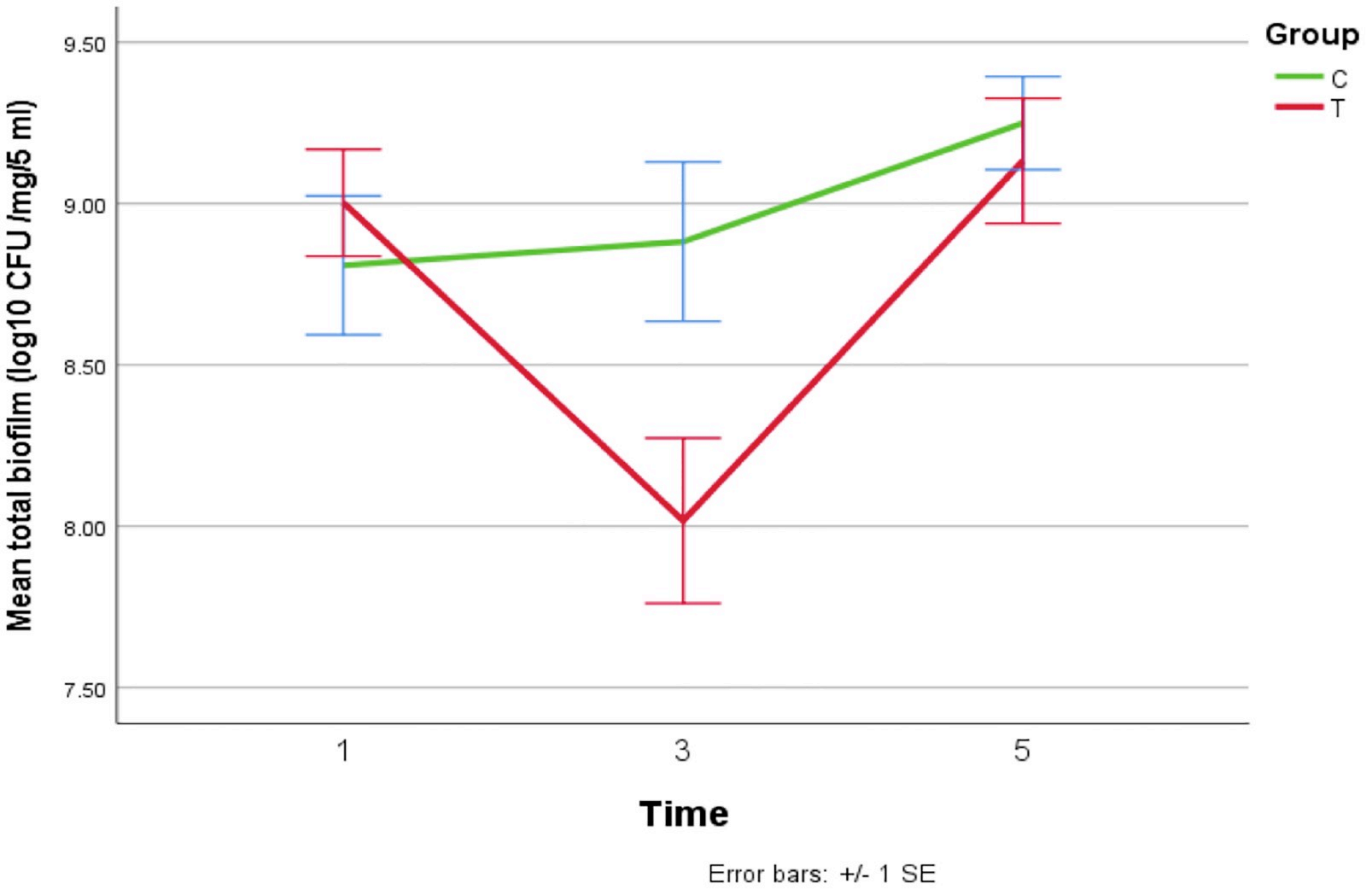
Effect of GIC containing *tt*-farnesol on viability of biofilm of *S*. *mutans* expressed as average values for log10 of the numbers of CFU/milliliter versus day of treatment. T–GIC + *tt*-farnesol; C- GIC (control).

Biofilm bacterial viability (CFU/mg of biofilm). The viability of the *S*. *mutans* was assessed by determination of CFU/mg of biofilm. Fig 1 demonstrates the mean bacterial counts after biofilm assays over time and between treatment groups. A slight increase of CFU was noted in control group over time but means hovered in a similar range of 10^9^ (p>0.05). By contrast, the experimental group started at 10^9^, decreased to 10^8^ after 3 days (p<0.05) and then returned to baseline at day 5, possibly indicating a short-term antibacterial effect on GIC containing *tt*-farnesol, even though there is no statistical difference.

**Fig 2:**
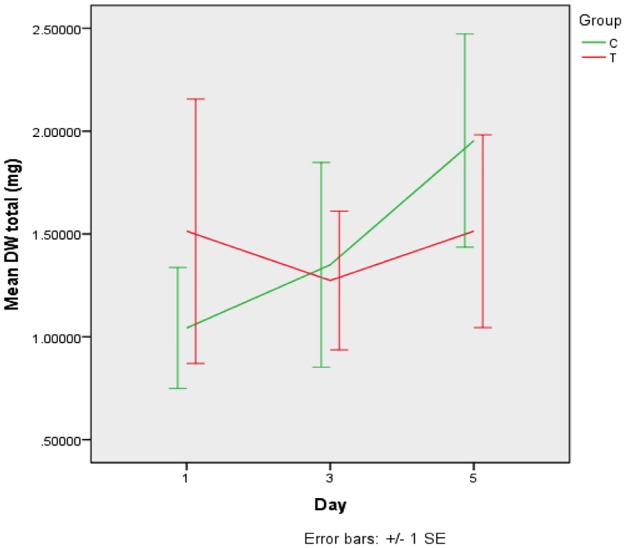
Means (±SD) of dry weight in *S*. *mutans* biofilms according to treatments after 1, 3 and 5 days of formation. T–GIC + *tt*-farnesol; C- GIC (control).

Biomass (dry weight). The dry weight of the *S*. *mutans* biofilm for each group and time point is shown Fig 2. Mean dry weight varied between 1 and 2 mg throughout and analysis failed to show differences as a function of treatment or time.

**Fig 3:**
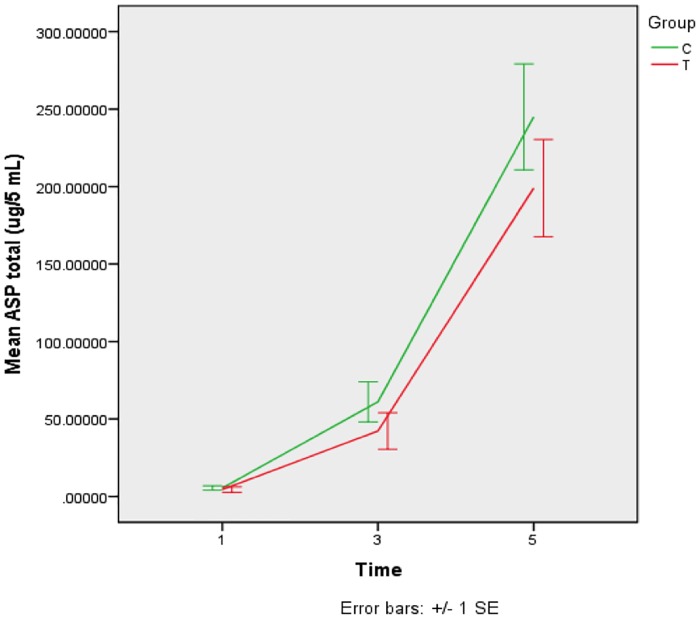
Means (±SD) of alkali soluble polysacchrides (ASP) concentration (μg/mg dry weight) in *S*. *mutans* biofilms according to treatments after 1, 3 and 5 days of formation. T–GIC + *tt*-farnesol; C- GIC (control).

**Fig 4:**
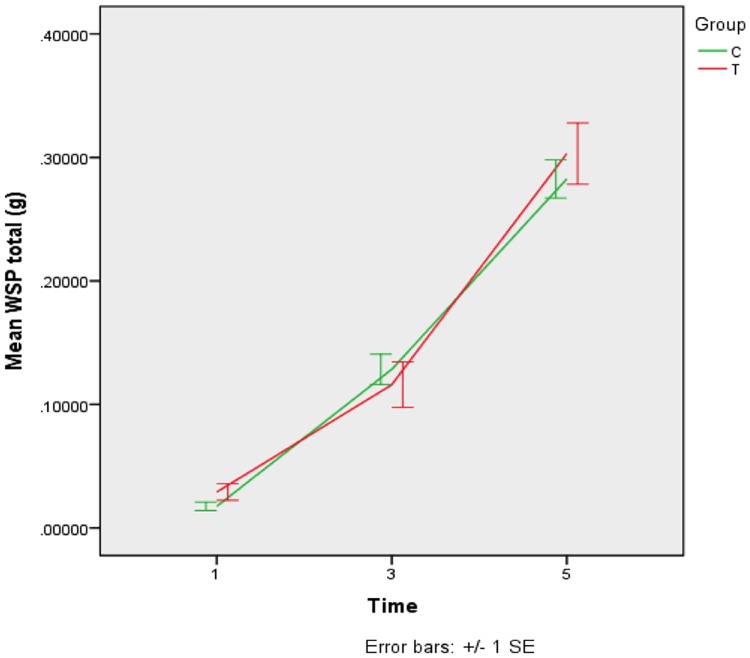
Means (±SD) of water soluble polysaccharides (WSP) concentration (g) in *S*. *mutans* biofilms according to treatments after 1, 3 and 5 days of formation. T–GIC + *tt*-farnesol; C- GIC (control).

Biochemical composition (ASP/WSP). The biochemical composition of the *S*. *mutans* biofilm for each group and time point is shown in Figs 3 and 4. Mean ASP and WSP concentrations increased after 3 and 5 days for both groups (p<0.05). There were no statistical differences between the experimental and control group at any time point (p>0.05).

**Fig 5:**
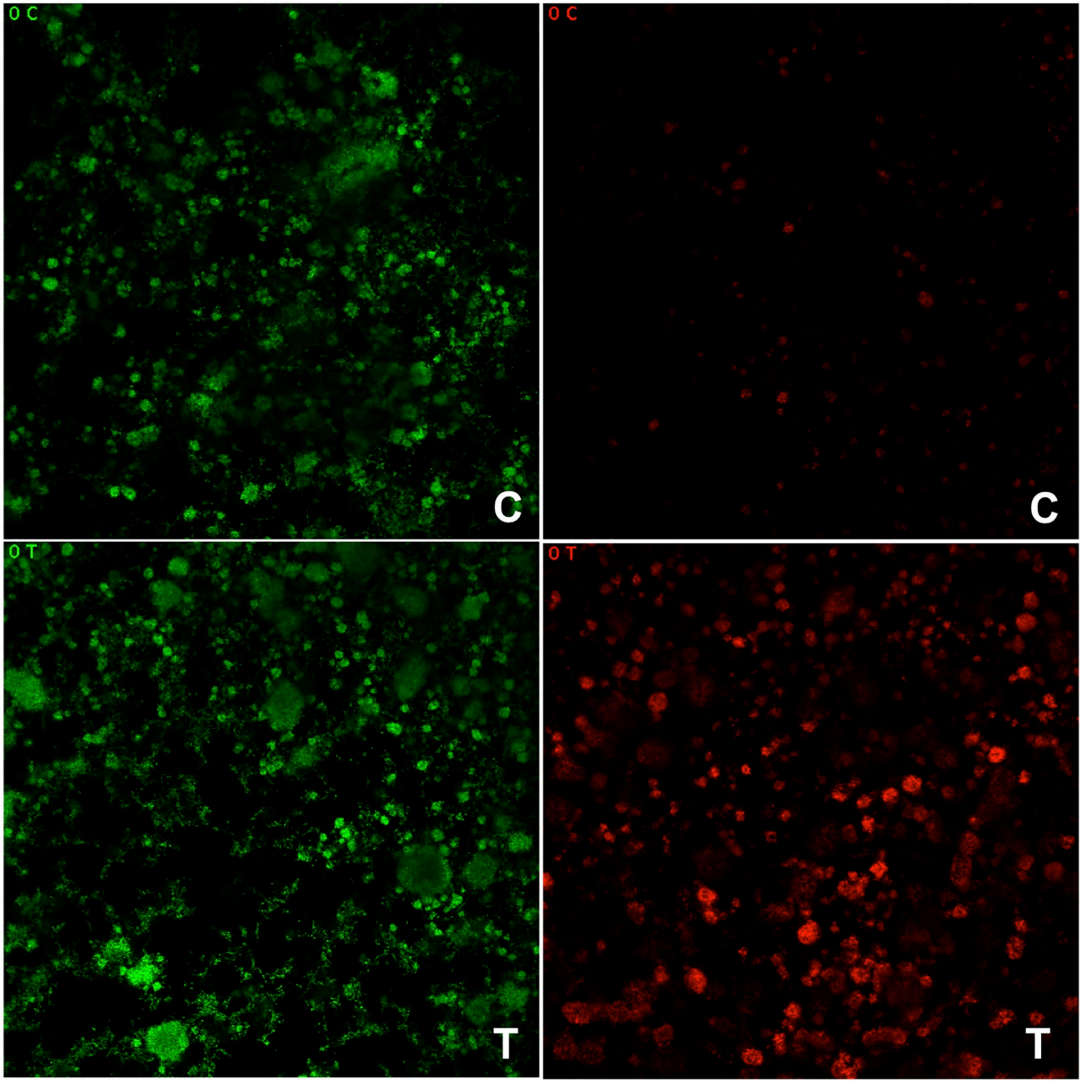
Representative images of *S*. *mutans* biofilms viability after treatment with GIC containing (T) or not (C) *tt-*farnesol visualized by confocal laser scanning microscopy (CLSM). Viable bacteria are stained green and non-viable bacteria are stained red color. It can be noted that both treatment and control slightly affected the biofilm formation but T was more effective on viability of bacteria.

Biofilm morphology analysis. CLSM images showed *S*. *mutans* biofilm on the GIC specimen surface with viable and non-viable colonies (green and red, respectively) and non-stained (black) bubble-like structures within the biofilm architecture (Fig 5, S1–S4 Figs). Non-viable bacteria (red colonies) were seen on the lower regions of the biofilm (specimen surface) after treatments, although in lower number and without a gradual increase of them over the days. Both experimental and control groups demonstrated predominant viable bacteria at day 5, evidenced by almost all colonies remained green, indicating that *S*. *mutans* were viable during the period evaluated.

**Fig 6:**
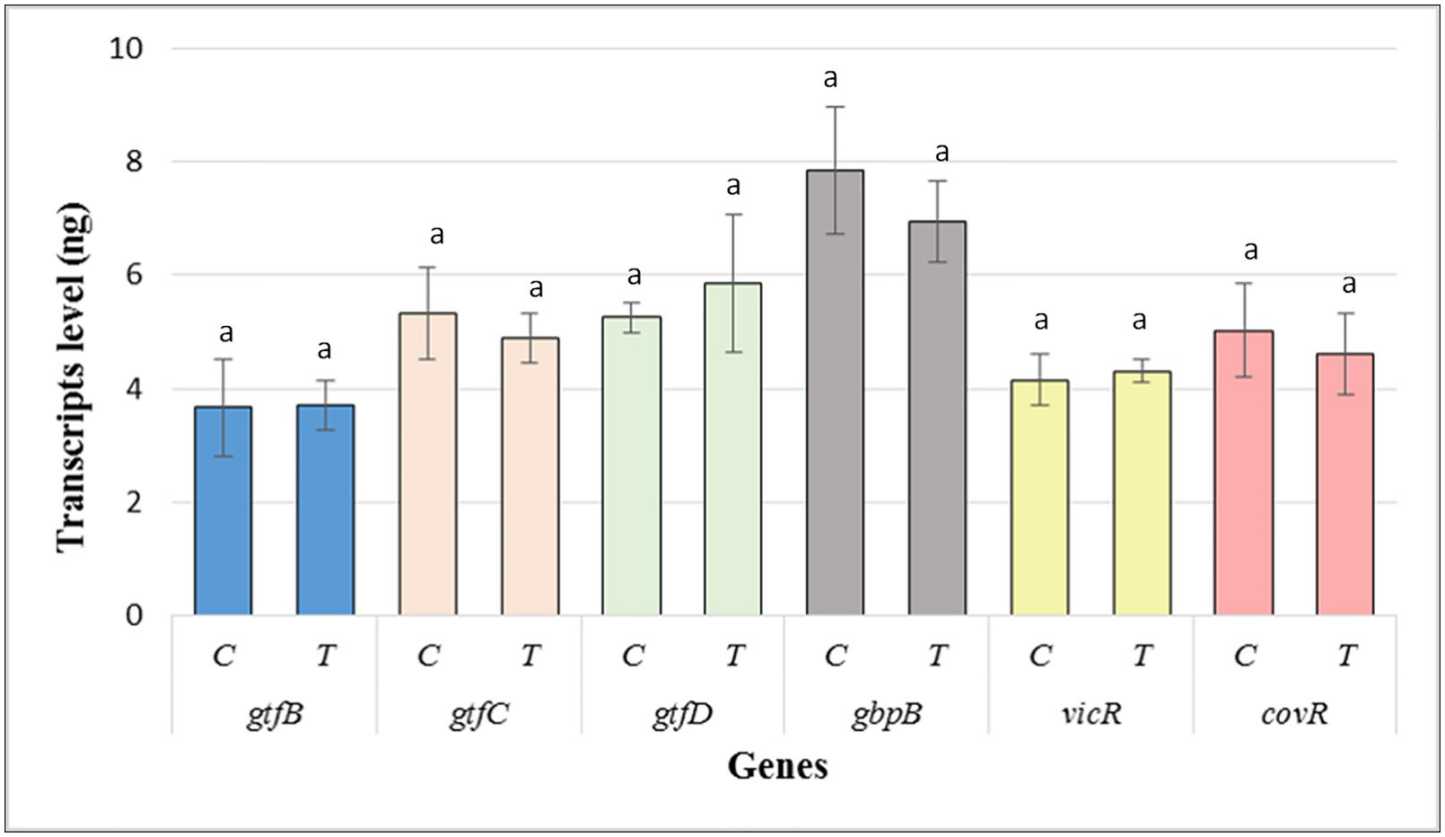
Means (±SD) of the gene expression values in transcript levels (ng) after 24 h of biofilm formation. T–GIC + *tt-*farnesol; C- GIC (control).

Gene expression (RT-qPCR). Gene analysis revealed low variation on their expression after 24 h of biofilm formation without significant difference between treatments (p<0.05), showing that the *tt-*farnesol did not modulate the *S*. *mutans* virulence (Fig 6).

### MTT test

Considering the negative control as 100% of cell viability, the percentage of cell viability observed for experimental and control groups was 90% (±7.3) and 61% (±10.4), respectively. After 24 h of treatments, only GIC reduced considerably the cell viability of HaCat cells (p < 0.05).

**Fig 7:**
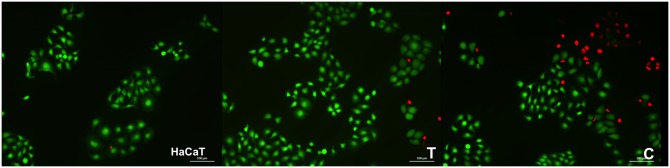
Illustrative images of HaCaT cells exposed for 24 h to GIC containing (A) or not (B) *tt*-farnesol analyzed by using Live/Dead assay. Green cells correspond to live cells whereas dead cells are stained in red.

Morphological characteristics of normal keratinocytes were observed for both groups, in which the intense green tone predominates, displaying cell viability after treatments. GIC showed more red fluorescence than GIC + *tt-*farnesol, evidencing that the control group was more cytotoxic than the experimental group (Fig 7).

### Physical properties

The means and standard deviations of the values obtained for physical testing are shown in Table 2. GIC samples containing *tt*-farnesol were harder than controls, but no differences were observed between the groups for compressive strength, diametral tensile strength or roughness (p>0.05).

**Table 2:** Roughness, hardness, compressive and diametral tensile strength of GIC containing or not *tt*-farnesol (mean ± standard deviation).

Groups			Mechanical properties*	
	Roughness (μM)	Hardness (KHN)	Compressive Strength (MPa)	Diametral Tensile Strength (MPa)
CIV (control)	0.7 ± 0.1 ^a^	44.2 ± 8.0 ^a^	24.0 ± 8.9 ^a^	18.0 ± 5.5 ^a^
CIV + *tt*-farnesol	0.8 ± 0.1 ^a^	72.4 ± 6.5 ^b^	28.5 ± 8.3 ^a^	14.0 ± 3.4 ^a^

*For each assay, different lower-case letters indicate mean differences (p<0.05).

### Raman spectroscopy

**Fig 8:**
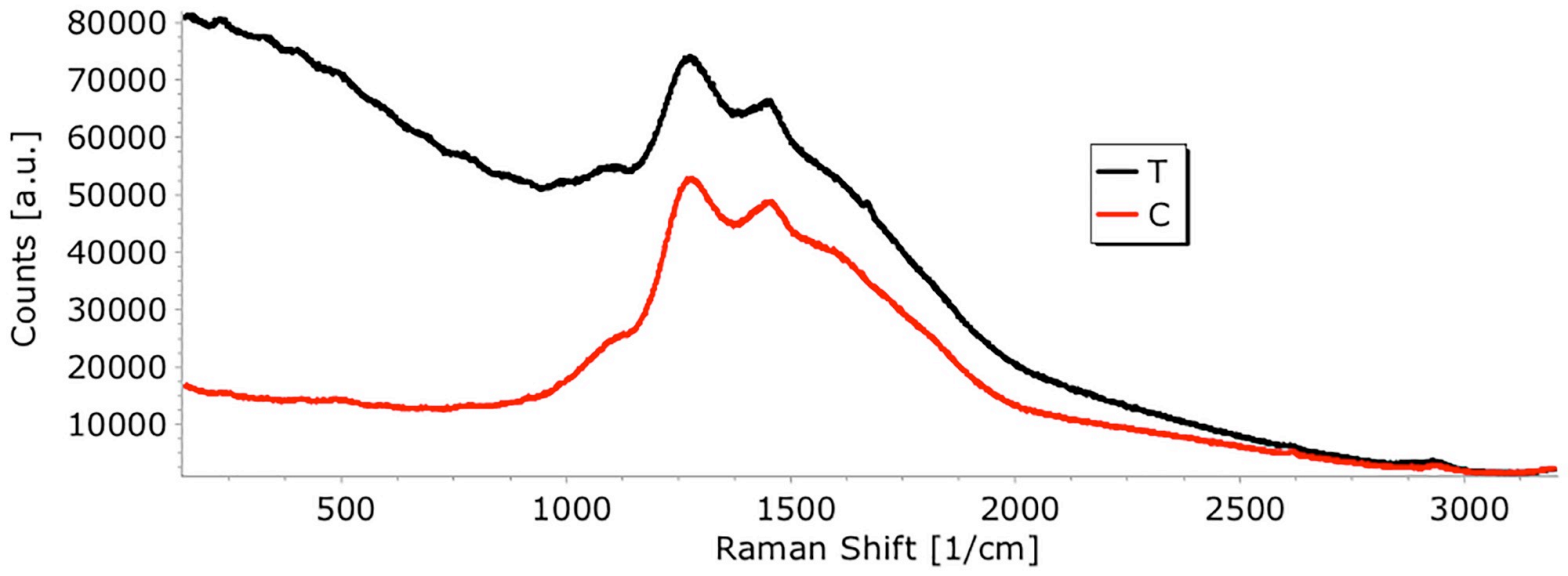
Raman spectra of *tt*-farnesol in the 150 to 1000 cm^−1^ range. T–GIC + *tt*-farnesol; C- GIC (control).

The Raman spectra of *tt*-farnesol in GIC and GIC control are shown in Fig 8. Fluorescence in the spectrum of the tt-farnesol in the GIC sample is observed in the region of 150 to 1000 cm^-1^. In the mentioned region of this sample it is still possible to identify some bands besides evidencing the appearance of a peak in 1673 cm^-1^ all this indicating a change in the chemical environment of the GIC when the compound is present (Fig 8).

## Discussion

Considering that dental caries is still one of the most frequent worldwide conditions [24], it is reasonable to improve the existing therapies for the treatment and prevention of cavity lesions. The selection of a dental material involves their physical, mechanical and biological properties as well as their clinical application. As GIC is the material of first choice for high caries activity patients [25], the improvement of their antimicrobial property is supported by the great potential for clinical indication besides other advantageous properties performance [1]. Likewise, since natural products have been in vogue due to their pharmacological and biological activities [4], the idea of incorporating a natural compound to GIC was backed up by the possibility to characterize a safe and also more effective restorative material.

Developing a novel dental material has long been a challenge due to countless assays required prior clinical use [5]. As *S*. *mutans* has a key role in the process of dental caries [8], several studies have focused the attention on that pathogen, exploring initial antimicrobial assays.

One of the hypotheses preceding the beginning of this study was that *S*. *mutans* biofilm formation would be disturbed by the presence of natural compound added to the restorative material. The *tt*-farnesol is known as an effective natural antimicrobial against planktonic and biofilm-associated *S*. *mutans* cells [26]. Our study also demonstrated that the incorporation of *tt*-farnesol to the GIC had the best antibacterial activity against free-floating planktonic bacteria (*S*. *mutans*, *L*. *casei*, *L*. *acidophilus* and *A*. *naeslundii*). Thus, the first hypothesis of this study was accepted. However, we found here that there were important differences in the antimicrobial response of planktonic and biofilm cells. Herein, the viability of the biofilms was affected only 3 days after treatment although the reduction of bacteria was irrelevant in the end of a week. This suggests that the antimicrobial activity of GIC containing *tt*-farnesol is effective on planktonic cells after 24 h and it also has progressive increasing up to 3 days of treatment. That is an interesting observation as the experimental material could establish an anticariogenic environment around restorations in early stages of caries formation and thus, would somehow disturb the microorganism adhesion to the surface and also avoid the development of new carious lesions on the tooth-restoration interface [2].

Despite the progressive increasing of the antimicrobial activity over the days, probably the *tt-*farnesol activity was restricted to the GIC surface, without substantial impact on mature biofilm. It is known that bacterial biofilms are complex communities of microorganisms embedded within an extra-cellular matrix [27]. Regarding the complexity of the biofilm, in this study, one of the possible reasons for the lack of activity of the treatment on mature biofilm could be the bacterial virulence. Since *S*. *mutans* is an acidogenic and aciduric species [28] the low-pH environment arouses the ability to form biofilm [27]. Therefore, it is clear that the pathogenesis of dental caries is dependable of the production of organic acids on dental biofilm, mainly lactic acid, by bacteria with acidogenic and aciduric potentials like the *S*. *mutans* [8]. As the medium pH in this study was 4.5, the therapy for oral biofilm control was useless.

Another evidence of the existing but surface-limited antimicrobial effect of the experimental material is shown by cell morphology. As expected, CSLM reveal some non-viable cells after 5 days of treatment. Those cells probably were sub-populations of bacteria on the surface of material that were directly exposed to the treatment. Interestingly, similar results were found by Hu et al. [29] that evaluated the association of the same conventional GIC with the flavonoid epigallocatechin-3-gallate (EGCG). Those authors observed a significantly decreased of planktonic cells after 24 h of treatment, without inhibition zones for GIC [29].

Based on cellular findings and considering that the virulence of *S*. *mutans* depends on the environmental conditions of the biofilm [27], we expanded the current study for the molecular level. In particular, as the incorporation of *tt*-farnesol into GIC affected only planktonic cells but not mature biofilm or the *S*. *mutans* expression profile we strongly believe that the compound was inactivated after the setting time of material. Setting of GIC occurs in two steps, including the initial setting that takes about 10 min and the second step, which is slow and continues up to 24 h. This process could perhaps be responsible for the antimicrobial effect of material in the first hours as well as the absence of inhibition after 24 h of treatment [1]. The anti-caries activity and the capacity to modulate the *S*. *mutans* virulence of that terpene were already broadly disclosed [14, 15, 30] as their antimicrobial spectra after incorporation into other dental material [31]. However, it seems that the association of *tt*-farnesol with other compounds [14] or materials as GIC promote a synergic effect on their biological properties; thus, a little or absent inhibitory activity is shown on the composition, structure and physiology of the biofilm matrices, rejecting then, our second hypothesis. Undoubtedly, in this biofilm model, the use of natural compounds associated with a dental material resulted in no apparent reduction in total biomass and absence of variation of the biochemical composition of the biofilm as well as a lack of modulation on *S*. *mutans* gene expression. Till date, some studies have been undertaken to assess the biological and physical properties of antimicrobial-containing GIC. However, no other study had been performed in order to evaluate the anti-caries potential of the incorporation of natural products to that GIC. Due to the lack of literature background, we cannot compare our results with other studies.

The idea of incorporating a natural product into GIC focused on the improvement of their antimicrobial activity. Furthermore, the incorporation of a bioactive can affect the physical properties of the GIC, herein we demonstrated that is possible to modify their composition without jeopardize important physical properties. Whereas the compressive strength is a basic material property that reproduces the resistance of the material to masticatory force, the diametral tensile strength intend to simulate the resistance to axial forces in opposite directions [2]. Likewise, the surface characteristics are also a determinant factor for clinical recommendation of a dental material as they can influence on polishing, scratching as well as the resistance to load application [2]. While the Knoop hardness is a sensitive surface parameter that assesses the resistance to plastic deformation [2], the roughness of the material influences straight on the bacterial adhesion [32]. Herein all physical properties of GICs but hardness, remained unchanged after the incorporating of the *tt*-farnesol. Those are interesting findings that could indicate the success of the GIC containing *tt*-farnesol in oral environment if we consider their toughness to forces, tension, deformation, wear as well as the control of early adhesion of bacteria on surface. Taking all together, those findings suggest no significant modifications in the powder/liquid ratios by adding a bioactive.

Corroborating with the physical findings, the Raman spectral features characterized the chemical change of GIC after the incorporation of the bioactive. Raman spectroscopy is a well-established simple and nondestructive analysis, which gives details about the chemical and structural composition of the material [23]. However, it seems that the association between *tt*-farnesol and some GIC component might affects totally or partially the chemical bonds as no greater peak was observed in the Raman spectral. Although the exact mechanism of that bioactive compound interaction needs further explanation to support whether occurs their decomposition or inactivation, the results herein reinforce the premise that the chemical variation occurred but it was not enough to modify all physical properties or biofilm structure.

As the importance of physical and chemical properties, the understanding of the biological safety of an experimental material cannot be neglect during a characterization. Surprisingly, MTT assay showed that the most biocompatible material was the GIC containing *tt-*farnesol, with a little effect on cell metabolism, evidencing a protective effect on keratinocytes. This finding was unexpected and shows that the incorporation of a bioactive into GIC might decrease their toxicity. These results are in agreement with previous studies, which observed that the incorporation of different antimicrobial substances could be safe and useful in Dentistry [16,17,22] and were confirmed by Live/Dead assay as well, which demonstrated a high number of viable cells after 24h of treatments. Therefore, the third and forth hypotheses of the current study were accepted.

The main findings of the current study were the upgrading of antimicrobial activity of GIC, keeping their basic properties. While the inhibitory effect was shown by contact not affecting the biofilm matrix and physiology, the experimental GIC could still be clinically acceptable considering the material weaknesses, since their hardness was increased by the incorporation of the bioactive, as well as their greater biocompatibility to human cells.

Even though the incorporation of *tt*-farnesol didn’t interfere negatively either with the physical properties or the toxicity, further studies need to be done with higher doses or different mechanisms of incorporation to improve the material antibiofilm activity.

## Supporting information

S1 Fig Biofilm (dead cells) after treatment with GIC. (AVI)

S2 Fig Biofilm (live cells) after treatment with GIC. (AVI)

S3 Fig Biofilm (dead cells) after treatment with GIC + *tt*-farnesol. (AVI)

S4 Fig Biofilm (live cells) after treatment with GIC + *tt*-farnesol. (AVI)

S1 Text Statistical analysis (agar diffusion test). (PDF)

S2 Text Statistical analysis (*covR* gene). (PDF)

S3 Text Statistical analysis (*gtfB* gene). (PDF)

S4 Text Statistical analysis (*gtfC* gene). (PDF)

S5 Text Statistical analysis (*gtfD* gene). (PDF)

S6 Text Statistical analysis (*gbpD* gene). (PDF)

S7 Text Statistical analysis (*vicR* gene). (PDF)

S8 Text Statistical analysis (MTT assay). (PDF)

S9 Text Statistical analysis (Physical properties). (PDF)

S10 Text Statistical analysis (ASP analysis). (PDF)

S11 Text Statistical analysis (CFU analysis). (PDF)

S12 Text Statistical analysis (DW analysis). (PDF)

S13 Text Statistical analysis (WSP analysis). (PDF)

## References

[1] Sidhu SK, Nicholson JW. A Review of Glass-Ionomer Cements for Clinical Dentistry. J Funct Biomater. 2016; 7: 16. 10.3390/jfb7030016 PMC5040989 27367737

[2] Wang SP, Ge Y, Zhou XD, Xu HHK, Weir MD, Zhang KK, et al Effect of anti-biofilm glass–ionomer cement on Streptococcus mutans biofilms. Int J Oral Sci. 2016; 8: 76–83. 10.1038/ijos.2015.55 27357319 PMC4932770

[3] American Academy of Pediatric Dentistry. Pediatric Restorative Dentistry. Reference Manual. 2016; 40: 18–19. https://www.aapd.org/research/oral-health-policies—recommendations/pediatric-restorative-dentistry/

[4] Newman DJ, Cragg GM. Natural Products as Sources of New Drugs from 1981 to 2014. J Nat Prod. 2016; 79: 629–661. 10.1021/acs.jnatprod.5b01055 26852623

[5] Freires IA, Rosalen PL. How Natural Product Research has Contributed to Oral Care Product Development? A Critical View. Pharm Res. 2016; 33: 1311–1317. 10.1007/s11095-016-1905-5 26975359

[6] Koo K. Strategies to enhance the biological effects of fluoride on dental biofilms. Adv Dent Res. 2008; 20: 17–21. 10.1177/154407370802000105 18694872

[7] Faustova MO, Ananieva MM, Basarab YO, Dobrobolska OV, Vovk IM, Loban’ GA. Bacterial factors of cariogenicity (literature review). Wiad Lek. 2018; 71: 378–382. 29786589

[8] Bowen WH, Burne RA, Wu H, Koo H. Oral Biofilms: Pathogens, Matrix, and Polymicrobial Interactions in Microenvironments. Trends Microbiol. 2018; 26: 229–242. 10.1016/j.tim.2017.09.008 29097091 PMC5834367

[9] Pitts NB, Zero DT, Marsh PD, Ekstrand K, Weintraub JA, Ramos-Gomez F, et al Dental caries. Nat Rev Dis Primers. 2017; 25: 17030. 10.1038/nrdp.2017.30 28540937

[10] Weber K, Delben J, Bromage TG, Duarte S. Comparison of SEM and VPSEM imaging techniques with respect to Streptococcus mutans biofilm topography. FEMS Microbiol Lett 2014; 350: 175–179. 10.1111/1574-6968.12334 24261820

[11] Neel EAA, Aljabo A, Strange A, Ibrahim A, Coathup M, Young AM, et al Demineralization-remineralization dynamics in teeth and bone. Int J Nanomedicine. 2016; 11: 4743–4763. 10.2147/IJN.S107624 27695330 PMC5034904

[12] Pedraza MCC, Novais TF, Faustoferri RC, Quivey RG Jr., Terekhov A, Hamaker BR, et al Extracellular DNA and lipoteichoic acids interact with exopolysaccharides in the extracellular matrix of *Streptococcus mutans* biofilms Biofouling. 2017; 33: 722–740. 10.1080/08927014.2017.1361412 28946780 PMC5929139

[13] Abbasi AJ, Mohammadi F, Bayat M, Gema SM, Ghadirian H, Seifi H, et al Applications of Propolis in Dentistry: A Review. Ethiop J Health Sci. 2018; 28: 505–512. 10.4314/ejhs.v28i4.16 30607063 PMC6308739

[14] Rocha GR, Florez Salamanca EJ, de Barros AL, Lobo CIV, Klein MI. Effect of *tt*-farnesol and myricetin on in vitro biofilm formed by *Streptococcus mutans* and *Candida albicans*. BMC Complement Altern Med. 2018; 18: 61 10.1186/s12906-018-2132-x 29444673 PMC5813409

[15] Jeon JG, Klein MI, Xiao J, Gregoire S, Rosalen PL, Koo H. Influences of naturally occurring agents in combination with fluoride on gene expression and structural organization of *Streptococcus mutans* in biofilms. BMC Microbiol. 2009; 9: 228 10.1186/1471-2180-9-228 19863808 PMC2774857

[16] de Castilho AR, Duque C, Negrini TC, Sacono NT, de Paula AB, de Souza Costa CA, et al In vitro and in vivo investigation of the biological and mechanical behaviour of resin-modified glass-ionomer cement containing chlorhexidine. J Dent. 2013; 41: 155–163. 23123495 10.1016/j.jdent.2012.10.014

[17] de Castilho AR, Duque C, Negrini TC, Sacono NT, de Paula AB, Sacramento PA, et al Mechanical and biological characterization of resin-modified glass-ionomer cement containing doxycycline hyclate. Arch Oral Biol. 2012; 57: 131–138. 10.1016/j.archoralbio.2011.08.009 21920494

[18] de Castilho AR, Duque C, Kreling PF, Pereira JA, Paula AB, Sinhoreti MAC, et al Doxycycline-containing glass ionomer cement for arresting residual caries: an in vitro study and a pilot trial. J Appl Oral Sci. 2018; 26: e20170116 10.1590/1678-7757-2017-0116 29742263 PMC5933828

[19] de Sousa DL, Lima RA, Zanin IC, Klein MI, Janal MN, Duarte S. Effect of Twice-Daily Blue Light Treatment on Matrix-Rich BiofilmDevelopment. PLoS One. 2015; 10: e0131941 10.1371/journal.pone.0131941 26230333 PMC4521953

[20] de Souza Araújo IJ, de Paula AB, Bruschi Alonso RC, Taparelli JR, Innocentini Mei LH, Stipp RN, et al A novel Triclosan Methacrylate-based composite reduces the virulence of Streptococcus mutans biofilm. PLoS One. 2018; 13: e0195244 10.1371/journal.pone.0195244 29608622 PMC5880362

[21] Stipp RN, Boisvert H, Smith DJ, Höfling JF, Duncan MJ, Mattos-Graner RO. CovR and VicRK regulate cell surface biogenesis genes required for biofilm formation in Streptococcus mutans. PLoS One. 2013; 8: e58271 10.1371/journal.pone.0058271 23554881 PMC3595261

[22] Duque C, Aida KL, Pereira JA, Teixeira GS, Caldo-Teixeira AS, Perrone LR, et al In vitro and in vivo evaluations of glass-ionomer cement containing chlorhexidine for Atraumatic Restorative Treatment. J Appl Oral Sci. 2017; 25: 541–550. 10.1590/1678-7757-2016-0195 29069152 PMC5804391

[23] Dainezi VB, Iwamoto AS, Martin AA, Soares LE, Hosoya Y, Pascon FM, et al Molecular and morphological surface analysis: effect of filling pastes and cleaning agents on root dentin. J Appl Oral Sci. 2017; 25: 101–111. 10.1590/1678-77572016-0053 28198982 PMC5289406

[24] Lagerweij MD, van Loveren C. Declining Caries Trends: Are We Satisfied? Curr Oral Health Rep. 2015; 2: 212–217. 10.1007/s40496-015-0064-9 26523247 PMC4623064

[25] Troca VBPB, Fernandes KBP, Terrile AE, Marcucci MC, de Andrade FB, Wang L. Effect of green propolis addition to physicalmechanical properties of glass ionomer cements. J Appl Oral Sci. 2011; 19: 100–105. 10.1590/S1678-77572011000200004 21552709 PMC4243746

[26] Libério SA, Pereira AL, Araújo MJ, Dutra RP, Nascimento FR, Monteiro-Neto V, et al The potential use of propolis as a cariostatic agent and its actions on mutans group streptococci. J Ethnopharmacol. 2009; 125: 1–9. 10.1016/j.jep.2009.04.047 19422903

[27] Krzyściak W, Jurczak A, Kościelniak D, Bystrowska B, Skalniak A. The virulence of Streptococcus mutans and the ability to form biofilms. Eur J Clin Microbiol Infect Dis. 2014; 33: 499–515. 10.1007/s10096-013-1993-7 24154653 PMC3953549

[28] Takahashi N, Nyvad B. The role of bacteria in the caries process: ecological perspectives. J Dent Res. 2011; 90: 294–303. 10.1177/0022034510379602 20924061

[29] Hu J, Du X, Huang C, Fu D, Ouyang X, Wang Y. Antibacterial and physical properties of EGCG-containing glass ionomer cements. J Dent. 2013; 41: 927–934. 10.1016/j.jdent.2013.07.014 23911600

[30] Cao L, Zhang ZZ, Xu SB, Ma M, Wei X. Farnesol inhibits development of caries by augmenting oxygen sensitivity and suppressing virulence-associated gene expression in Streptococcus mutans. J Biomed Res. 2017; 31: 333–343. 10.7555/JBR.31.20150151 28808205 PMC5548994

[31] André CB, Rosalen PL, Galvão LCC, Fronza BM, Ambrosano GMB, Ferracane JL, et al Modulation of Streptococcus mutans virulence by dental adhesives containing anti-caries agents. Dent Mater. 2017; 33: 1084–1092. 10.1016/j.dental.2017.07.006 28774430

[32] Bayrak GD, Sandalli N, Selvi-Kuvvetli S, Topcuoglu N, Kulekci G. Effect of two different polishing systems on fluoride release, surfaceroughness and bacterial adhesion of newly developed restorative materials. J Esthet Restor Dent. 2017; 12: 424–434. 10.1111/jerd.12313 28618104

