# Correction: Trans,trans-farnesol, an antimicrobial natural compound, improves glass ionomer cement properties

**DOI:** 10.1371/journal.pone.0259549

**Published:** 2021-10-28

**Authors:** Aline Rogéria Freire de Castilho, Pedro Luiz Rosalen, Isaac Jordão de Souza Araújo, Igor Lebedenco Kitagawa, Cecilia Atem Gonçalves de Araújo Costa, Malvin N. Janal, Marcelo Corrêa Alves, Simone Duarte, Paulo Noronha Lisboa Filho, Rafael Nobrega Stipp, Regina Maria Puppin-Rontani

There is an error in Table 2. The authors named the groups “CIV (control)” and “CIV + *tt*-farnesol” in error instead of “GIC (control)” and “GIC + *tt*-farnesol.” Please see the correct Table 2.

**Table 2:** Roughness, hardness, compressive and diametral tensile strength of GIC containing or not *tt*-farnesol (mean ± standard deviation).

Groups			Mechanical properties*	
	Roughness (μM)	Hardness (KHN)	Compressive Strength (MPa)	Diametral Tensile Strength (MPa)
GIC (control)	0.7 ± 0.2 ^a^	44.2 ± 8.0 ^a^	24.0 ± 8.9 ^a^	18.0 ± 5.5 ^a^
GIC + *tt*-farnesol	0.8 ± 0.1 ^a^	72.4 ± 6.5 ^b^	28.5 ± 8.3 ^a^	14.0 ± 3.1 ^a^

*For each assay, different lower-case letters indicate mean differences (p<0.05).
