# Supplementary material for: Trans,trans-farnesol, an antimicrobial natural compound, improves glass ionomer cement properties
Source: PLoS One. 2019 Aug 20;14(8):e0220718. doi: 10.1371/journal.pone.0220718 (PMC6701760; doi:10.1371/journal.pone.0220718)
Supplement: S1 Text — (PDF) [file pone.0220718.s005.pdf]

Model Information	
Data Set	D.DADOS_AC_BIO
Response Variable	t_hi_act = hi_act + 0.5;
Response Distribution	Lognormal
Link Function	Identity
Variance Function	Default
Variance Matrix	Diagonal
Estimation Technique	Restricted Maximum Likelihood
Degrees of Freedom Method	Residual

Class Level Information		
Class	*Levels*	*Values*
tratamento	3	chx civ 2 tt mic

Number of Observations Read	30
Number of Observations Used	14

Dimensions	
Covariance Parameters	1
Columns in X	4
Columns in Z	0
Subjects (Blocks in V)	1
Max Obs per Subject	14

Optimization Information	
Optimization Technique	None
Parameters	4
Lower Boundaries	1
Upper Boundaries	0
Fixed Effects	Not Profiled

Fit Statistics	
-2 Res Log Likelihood	-42.04
AIC (smaller is better)	-34.04
AICC (smaller is better)	-27.37
BIC (smaller is better)	-32.45
CAIC (smaller is better)	-28.45

HQIC (smaller is better)	-35.04
Pearson Chi-Square	0.01
Pearson Chi-Square / DF	0.00

Type III Tests of Fixed Effects				
Effect	*Num DF*	*Den DF*	*F Value*	*Pr > F*
tratamento	2	11	8680.27	<.0001

tratamento Least Squares Means					
Material	*Estimate*	*Standard Error*	*DF*	*t Value*	*Pr > |t|*
chx	1.4327	0.01685	11	85.05	<.0001
civ 2	-0.6931	0.01191	11	-58.19	<.0001
tt mic	1.3518	0.01305	11	103.60	<.0001

Differences of tratamento Least Squares Means Adjustment for Multiple Comparisons: Tukey-Kramer							
Material	*Material*	*Estimate*	*Standard Error*	*DF*	*t Value*	*Pr > |t|*	*Adj P*
chx	civ 2	2.1258	0.02063	11	103.04	<.0001	<.0001
chx	tt mic	0.08089	0.02131	11	3.80	0.0030	0.0076
civ 2	tt mic	-2.0450	0.01767	11	-115.75	<.0001	<.0001

Tukey-Kramer Grouping for tratamento Least Squares Means (Alpha=0.05)		
LS-means with the same letter are not significantly different.		
Material	*Estimate*	
chx	1.4327	A
tt mic	1.3518	B
civ 2	-0.6931	C

Material	*Halo de inibição - Actinomices*	*Resíduo*
tt mic	3.16	-0.054344
tt mic	3.17	-0.051615
civ 2	0.00	0.000000
civ 2	0.00	0.000000

civ 2	0.00	0.000000
chx	3.69	0.000000
chx	3.69	0.000000
tt mic	3.50	0.034487
tt mic	3.50	0.034487
tt mic	3.51	0.036984

Coeficiente de assimetria	*Coeficiente de curtose*	*W Shapiro-Wilk*	*Valor-p Ho:Normal*
-0.76771	0.99897	0.77166	.002274353

Analysis Variable : hi_act Halo de inibição - Actinomices

Material	*N Obs*	*Mean*	*Std Dev*	*Upper 95% CL for Mean*	*Lower 95% CL for Mean*
chx	9	3.69	0.00	.	.
civ 2	9	0.00	0.00	.	.
tt mic	12	3.37	0.19	3.60	3.14

Model Information

Data Set	D.DADOS_AC_BIO
Response Variable	t_hi_lc = hi_lc + 0.5;
Response Distribution	Lognormal
Link Function	Identity
Variance Function	Default
Variance Matrix	Diagonal
Estimation Technique	Restricted Maximum Likelihood
Degrees of Freedom Method	Residual

Class Level Information

Class	*Levels*	*Values*
tratamento	3	chx civ 2 tt mic

Number of Observations Read	30
Number of Observations Used	18

Dimensions

Covariance Parameters	1
Columns in X	4
Columns in Z	0
Subjects (Blocks in V)	1
Max Obs per Subject	18

Optimization Information	
Optimization Technique	None
Parameters	4
Lower Boundaries	1
Upper Boundaries	0
Fixed Effects	Not Profiled

Fit Statistics	
-2 Res Log Likelihood	-10.65
AIC (smaller is better)	-2.65
AICC (smaller is better)	1.35
BIC (smaller is better)	0.19
CAIC (smaller is better)	4.19
HQIC (smaller is better)	-2.68
Pearson Chi-Square	0.30
Pearson Chi-Square / DF	0.02

Type III Tests of Fixed Effects

Effect	*Num DF*	*Den DF*	*F Value*	*Pr > F*
tratamento	2	15	200.38	<.0001

tratamento Least Squares Means

Material	*Estimate*	*Standard Error*	*DF*	*t Value*	*Pr > |t|*
chx	0.9061	0.05791	15	15.65	<.0001
civ 2	-0.6931	0.05791	15	-11.97	<.0001
tt mic	0.4193	0.05791	15	7.24	<.0001

Differences of tratamento Least Squares Means
Adjustment for Multiple Comparisons: Tukey

Material	*Material*	*Estimate*	*Standard Error*	*DF*	*t Value*	*Pr > |t|*	*Adj P*
chx	civ 2	1.5992	0.08190	15	19.53	<.0001	<.0001
chx	tt mic	0.4868	0.08190	15	5.94	<.0001	<.0001
civ 2	tt mic	-1.1124	0.08190	15	-13.58	<.0001	<.0001

Tukey Grouping for tratamento Least Squares Means (Alpha=0.05)

LS-means with the same letter are not significantly different.		
Material	*Estimate*	
chx	0.9061	A
tt mic	0.4193	B
civ 2	-0.6931	C

Material	*Halo de inibição - L.casei*	*Resíduo*
chx	1.54	-0.19313
chx	1.54	-0.19313
chx	1.55	-0.18824
tt mic	0.89	-0.08999
tt mic	0.89	-0.08999
tt mic	1.09	0.04444
chx	2.23	0.09822
tt mic	1.39	0.21728
chx	2.64	0.23814
chx	2.64	0.23814

Coeficiente de assimetria	*Coeficiente de curtose*	*W Shapiro-Wilk*	*Valor-p Ho:Normal*
0.43727	-0.17336	0.90353	0.066225

Analysis Variable : hi_lc Halo de inibição - L.casei					
Material	*N Obs*	*Mean*	*Std Dev*	*Upper 95% CL for Mean*	*Lower 95% CL for Mean*
chx	9	2.02	0.55	2.60	1.45
civ 2	9	0.00	0.00	.	.
tt mic	12	1.03	0.19	1.23	0.83

Model Information	
Data Set	D.DADOS_AC_BIO
Response Variable	t_hi_la = hi_la + 0.5;
Response Distribution	Gamma
Link Function	Log
Variance Function	Default
Variance Matrix	Diagonal
Estimation Technique	Maximum Likelihood
Degrees of Freedom Method	Residual

Class Level Information		
Class	*Levels*	*Values*
tratamento	3	chx civ 2 tt mic

Number of Observations Read	30
Number of Observations Used	30

Dimensions	
Covariance Parameters	1
Columns in X	4
Columns in Z	0
Subjects (Blocks in V)	1
Max Obs per Subject	30

Optimization Information	
Optimization Technique	Newton-Raphson
Parameters in Optimization	4
Lower Boundaries	1
Upper Boundaries	0
Fixed Effects	Not Profiled

Iteration History					
Iteration	*Restarts*	*Evaluations*	*Objective Function*	*Change*	*Max Gradient*
0	*0*	4	51.891846084	.	31.04871
1	*0*	28	23.308140221	28.58370586	13.73637
2	*0*	5	21.927264917	1.38087530	11.02827
3	*0*	3	19.641750858	2.28551406	134.5021
4	*0*	3	19.376675684	0.26507517	28.44081
5	*0*	3	19.358861145	0.01781454	2.192774
6	*0*	3	19.358692585	0.00016856	0.017864
7	*0*	3	19.358692318	0.00000027	0.000072
8	*0*	3	19.358692318	0.00000000	9.991E-7

Convergence criterion (GCONV=1E-8) satisfied.

Fit Statistics	
-2 Log Likelihood	38.72

AIC (smaller is better)	46.72
AICC (smaller is better)	48.32
BIC (smaller is better)	52.32
CAIC (smaller is better)	56.32
HQIC (smaller is better)	48.51
Pearson Chi-Square	0.69
Pearson Chi-Square / DF	0.03

Type III Tests of Fixed Effects				
Effect	*Num DF*	*Den DF*	*F Value*	*Pr > F*
tratamento	2	27	914.94	<.0001

tratamento Least Squares Means					
Material	*Estimate*	*Standard Error*	*DF*	*t Value*	*Pr > |t|*
chx	1.5539	0.05127	27	30.31	<.0001
civ 2	-0.6931	0.05127	27	-13.52	<.0001
tt mic	2.1204	0.04440	27	47.76	<.0001

Differences of tratamento Least Squares Means Adjustment for Multiple Comparisons: Tukey-Kramer							
Material	*Material*	*Estimate*	*Standard Error*	*DF*	*t Value*	*Pr > |t|*	*Adj P*
chx	civ 2	2.2471	0.07250	27	30.99	<.0001	<.0001
chx	tt mic	-0.5664	0.06782	27	-8.35	<.0001	<.0001
civ 2	tt mic	-2.8135	0.06782	27	-41.49	<.0001	<.0001

Tukey-Kramer Grouping for tratamento Least Squares Means (Alpha=0.05)		
LS-means with the same letter are not significantly different.		
Material	*Estimate*	
tt mic	2.1204	A
chx	1.5539	B
civ 2	-0.6931	C

Material	*Halo de inibição - L.acidophilus*	*Resíduo*

tt mic	5.02	-0.33767
tt mic	5.30	-0.30407
chx	3.30	-0.19662
tt mic	6.37	-0.17568
chx	3.51	-0.15222
tt mic	8.81	0.11709
tt mic	9.75	0.22988
chx	5.45	0.25793
tt mic	10.42	0.31027
chx	5.82	0.33615

Coeficiente de assimetria	*Coeficiente de curtose*	*W Shapiro-Wilk*	*Valor-p Ho:Normal*
0.14074	0.70624	0.93794	0.080060

Analysis Variable : hi_la Halo de inibição - L.acidophilus					
Material	*N Obs*	*Mean*	*Std Dev*	*Upper 95% CL for Mean*	*Lower 95% CL for Mean*
chx	9	4.23	0.87	4.90	3.56
civ 2	9	0.00	0.00	.	.
tt mic	12	7.83	1.64	8.87	6.80

Model Information	
Data Set	D.DADOS_AC_BIO
Response Variable	t_hi_sm = hi_sm + 0.5;
Response Distribution	Gamma
Link Function	Log
Variance Function	Default
Variance Matrix	Diagonal
Estimation Technique	Maximum Likelihood
Degrees of Freedom Method	Residual

Class Level Information		
Class	*Levels*	*Values*
tratamento	3	chx civ 2 tt mic

Number of Observations Read	30
Number of Observations Used	22

Dimensions	
Covariance Parameters	1

Columns in X	4
Columns in Z	0
Subjects (Blocks in V)	1
Max Obs per Subject	22

Optimization Information	
Optimization Technique	Newton-Raphson
Parameters in Optimization	4
Lower Boundaries	1
Upper Boundaries	0
Fixed Effects	Not Profiled

Iteration History

Iteration	*Restarts*	*Evaluations*	*Objective Function*	*Change*	*Max Gradient*
0	*0*	4	52.765850373	.	19.39786
1	*0*	52	3.899696844	48.86615353	519.6886
2	*0*	3	3.8112265407	0.08847030	397.8355
3	*0*	3	3.6380764822	0.17315006	69.97781
4	*0*	3	3.6143573138	0.02371917	6.536343
5	*0*	3	3.6128817999	0.00147551	0.394418
6	*0*	3	3.6128507222	0.00003108	0.009193
7	*0*	3	3.6128505332	0.00000019	0.000231
8	*0*	3	3.6128505329	0.00000000	4.735E-6

Convergence criterion (GCONV=1E-8) satisfied.

Fit Statistics	
-2 Log Likelihood	7.23
AIC (smaller is better)	15.23
AICC (smaller is better)	17.58
BIC (smaller is better)	19.59
CAIC (smaller is better)	23.59
HQIC (smaller is better)	16.25
Pearson Chi-Square	0.06
Pearson Chi-Square / DF	0.00

Type III Tests of Fixed Effects

Effect	*Num DF*	*Den DF*	*F Value*	*Pr > F*
tratamento	2	19	2257.59	<.0001

tratamento Least Squares Means					
Material	*Estimate*	*Standard Error*	*DF*	*t Value*	*Pr > |t|*
chx	1.5686	0.02203	19	71.19	<.0001
civ 2	0.9047	0.01799	19	50.29	<.0001
tt mic	2.7269	0.02040	19	133.68	<.0001

Differences of tratamento Least Squares Means Adjustment for Multiple Comparisons: Tukey-Kramer							
Material	*Material*	*Estimate*	*Standard Error*	*DF*	*t Value*	*Pr > |t|*	*Adj P*
chx	civ 2	0.6639	0.02845	19	23.34	<.0001	<.0001
chx	tt mic	-1.1583	0.03003	19	-38.58	<.0001	<.0001
civ 2	tt mic	-1.8223	0.02720	19	-67.00	<.0001	<.0001

Tukey-Kramer Grouping for tratamento Least Squares Means (Alpha=0.05)		
LS-means with the same letter are not significantly different.		
Material	*Estimate*	
tt mic	2.7269	A
chx	1.5686	B
civ 2	0.9047	C

Material	*Halo de inibição - S. mutans*	*Resíduo*
tt mic	12.90	-0.12336
tt mic	14.00	-0.05140
tt mic	14.00	-0.05140
tt mic	14.00	-0.05140
civ 2	1.87	-0.04092
civ 2	2.07	0.04002
civ 2	2.07	0.04002
tt mic	16.00	0.07944
tt mic	16.20	0.09252
tt mic	16.40	0.10561

Coeficiente de assimetria	Coeficiente de curtose	W Shapiro-Wilk	Valor-p Ho:Normal
0.069468	-.001786301	0.95436	0.38420

Analysis Variable : hi_sm Halo de inibição - S. mutans					
Material	N Obs	Mean	Std Dev	Upper 95% CL for Mean	Lower 95% CL for Mean
chx	9	4.30	0.11	4.41	4.19
civ 2	9	1.97	0.10	2.05	1.90
tt mic	12	14.79	1.38	16.07	13.51

Obs	Material	Halo de inibição - Actinomices	Halo de inibição - L.casei	Halo de inibição - L.acidophilus	Halo de inibição - S. mutans
1	chx	3.69	1.54	3.51	4.40
2	chx	3.69	1.55	3.92	4.40
3	chx	3.69	1.54	3.30	4.40
4	chx	.	2.64	5.82	4.20
5	chx	.	2.64	3.63	4.20
6	chx	.	2.23	3.82	4.20
7	chx	.	.	4.28	.
8	chx	.	.	5.45	.
9	chx	.	.	4.34	.
10	civ 2	0.00	0.00	0.00	1.88
11	civ 2	0.00	0.00	0.00	1.95
12	civ 2	0.00	0.00	0.00	2.07
13	civ 2	0.00	0.00	0.00	1.87
14	civ 2	0.00	0.00	0.00	1.88
15	civ 2	0.00	0.00	0.00	1.88
16	civ 2	.	.	0.00	2.07
17	civ 2	.	.	0.00	2.07
18	civ 2	.	.	0.00	2.07
19	tt mic	3.17	0.96	8.32	16.40
20	tt mic	3.16	0.96	6.37	16.00
21	tt mic	3.50	0.89	7.35	16.20
22	tt mic	3.50	0.89	7.57	14.00
23	tt mic	3.51	1.39	8.22	14.00
24	tt mic	.	1.09	8.75	14.00
25	tt mic	.	.	9.75	12.90
26	tt mic	.	.	8.13	.
27	tt mic	.	.	8.81	.
28	tt mic	.	.	10.42	.

29	tt mic	.	.	5.30	.
30	tt mic	.	.	5.02	.
