# Supplementary material for: Trans,trans-farnesol, an antimicrobial natural compound, improves glass ionomer cement properties
Source: PLoS One. 2019 Aug 20;14(8):e0220718. doi: 10.1371/journal.pone.0220718 (PMC6701760; doi:10.1371/journal.pone.0220718)
Supplement: S2 Text — (PDF) [file pone.0220718.s006.pdf]

F1	false	2 Flags		covR	NTC	SYBR-Non	31.765	
F2	false	2 Flags		covR	NTC	SYBR-Non	30.763	
A1	true	Omitted by		covR	STANDARI	SYBR-None		
A2	true	Omitted by		covR	STANDARI	SYBR-None		
B1	false	No Flag		covR	STANDARI	SYBR-Non	12.497	12.502563
B2	false	No Flag		covR	STANDARI	SYBR-Non	12.508	12.502563
C1	false	No Flag		covR	STANDARI	SYBR-Non	17.097	17.175613
C2	false	No Flag		covR	STANDARI	SYBR-Non	17.254	17.175613
D1	false	No Flag		covR	STANDARI	SYBR-Non	22.318	22.440914
D2	false	No Flag		covR	STANDARI	SYBR-Non	22.564	22.440914
E1	false	No Flag		covR	STANDARI	SYBR-Non	27.242	27.11684
E2	false	No Flag		covR	STANDARI	SYBR-Non	26.991	27.11684
A3	false	No Flag	C1	covR	UNKNOWN	SYBR-Non	22.646	22.782417
A4	false	No Flag	C1	covR	UNKNOWN	SYBR-Non	22.919	22.782417
B3	false	No Flag	C2	covR	UNKNOWN	SYBR-Non	22.829	22.831856
B4	false	No Flag	C2	covR	UNKNOWN	SYBR-Non	22.835	22.831856
C3	false	No Flag	C3	covR	UNKNOWN	SYBR-Non	23.163	23.29437
C4	false	No Flag	C3	covR	UNKNOWN	SYBR-Non	23.426	23.29437
D3	false	No Flag	C4	covR	UNKNOWN	SYBR-Non	22.967	23.259377
D4	false	No Flag	C4	covR	UNKNOWN	SYBR-Non	23.552	23.259377
E3	false	No Flag	C5	covR	UNKNOWN	SYBR-Non	23.802	23.81942
E4	false	No Flag	C5	covR	UNKNOWN	SYBR-Non	23.837	23.81942
F3	false	No Flag	C6	covR	UNKNOWN	SYBR-Non	23.223	23.409794
F4	false	No Flag	C6	covR	UNKNOWN	SYBR-Non	23.597	23.409794
A5	false	No Flag	T1	covR	UNKNOWN	SYBR-Non	23.948	23.801601
A6	false	No Flag	T1	covR	UNKNOWN	SYBR-Non	23.656	23.801601
B5	false	No Flag	T2	covR	UNKNOWN	SYBR-Non	22.977	23.065378
B6	false	No Flag	T2	covR	UNKNOWN	SYBR-Non	23.154	23.065378
C5	true	Omitted by	T3	covR	UNKNOWN	SYBR-None		
C6	true	Omitted by	T3	covR	UNKNOWN	SYBR-None		
D5	false	No Flag	T5	covR	UNKNOWN	SYBR-Non	23.224	23.27745
D6	false	No Flag	T5	covR	UNKNOWN	SYBR-Non	23.331	23.27745
E5	true	Omitted by	T6	covR	UNKNOWN	SYBR-None		
E6	true	Omitted by	T6	covR	UNKNOWN	SYBR-None		

NaN		
NaN		
	300	
	300	
0.0079115	30	
0.0079115	30	
0.1108941	3	
0.1108941	3	
0.1745028	0.3	
0.1745028	0.3	
0.1773054	0.03	
0.1773054	0.03	
0.1925147	0.2508109	0.2357818
0.1925147	0.2207527	0.2357818
0.0041877	0.23023	0.2299108
0.0041877	0.2295915	0.2299108
0.1857617	0.1968447	0.1854381
0.1857617	0.1740316	0.1854381
0.4135827	0.215801	0.1899205
0.4135827	0.16404	0.1899205
0.0246219	0.1458827	0.1447015
0.0246219	0.1435203	0.1447015
0.2643814	0.1913993	0.1760104
0.2643814	0.1606215	0.1760104
0.2065263	0.136254	0.1462529
0.2065263	0.1562517	0.1462529
0.1253184	0.2148082	0.2062438
0.1253184	0.1976795	0.2062438
0.075952	0.1913187	0.1866203
0.075952	0.1819218	0.1866203

		Grupos	
		C	T
	1	0.24	0.15
	2	0.23	0.21
	3	0.19	
	4	0.19	
	5	0.14	0.19
	6	0.18	
Media		0.19	0.18
DP		0.03	0.03
	1	25.42	26.29
	2	25.84	25.45
	3	25.92	25.84
	4	26.07	
	5	26.40	25.34
	6	26.34	26.50
Media		26.00	25.88
DP		0.36	0.51
Normalizado			
	1	5.99	3.85
	2	5.94	5.25
	3	4.81	
	4	4.95	
	5	3.82	4.73
	6	4.64	
Media		5.03	4.61
DP		0.83	0.71
