# Supplementary material for: Trans,trans-farnesol, an antimicrobial natural compound, improves glass ionomer cement properties
Source: PLoS One. 2019 Aug 20;14(8):e0220718. doi: 10.1371/journal.pone.0220718 (PMC6701760; doi:10.1371/journal.pone.0220718)
Supplement: S3 Text — (PDF) [file pone.0220718.s007.pdf]

F1	false	One Flag		GTFB	NTC	SYBR-None	28.214	
F2	false	One Flag		GTFB	NTC	SYBR-None	28.601	
A1	false	No Flag		GTFB	STANDARD	SYBR-None	9.381	9.397606
A2	false	No Flag		GTFB	STANDARD	SYBR-None	9.414	9.397606
B1	false	No Flag		GTFB	STANDARD	SYBR-None	12.318	12.333685
B2	false	No Flag		GTFB	STANDARD	SYBR-None	12.349	12.333685
C1	false	No Flag		GTFB	STANDARD	SYBR-None	16.778	16.992077
C2	false	No Flag		GTFB	STANDARD	SYBR-None	17.206	16.992077
D1	false	No Flag		GTFB	STANDARD	SYBR-None	22.223	22.515844
D2	false	No Flag		GTFB	STANDARD	SYBR-None	22.809	22.515844
E1	false	One Flag		GTFB	STANDARD	SYBR-None	26.59	26.475498
E2	false	One Flag		GTFB	STANDARD	SYBR-None	26.361	26.475498
A3	false	No Flag	C1	GTFB	UNKNOWN	SYBR-None	22.859	23.0919
A4	false	No Flag	C1	GTFB	UNKNOWN	SYBR-None	23.325	23.0919
B3	false	No Flag	C2	GTFB	UNKNOWN	SYBR-None	22.776	22.8838
B4	false	No Flag	C2	GTFB	UNKNOWN	SYBR-None	22.991	22.8838
C3	false	No Flag	C3	GTFB	UNKNOWN	SYBR-None	23.309	23.265244
C4	false	No Flag	C3	GTFB	UNKNOWN	SYBR-None	23.222	23.265244
D3	false	No Flag	C4	GTFB	UNKNOWN	SYBR-None	24.211	23.95171
D4	false	No Flag	C4	GTFB	UNKNOWN	SYBR-None	23.692	23.95171
E3	false	No Flag	C5	GTFB	UNKNOWN	SYBR-None	24.029	24.090153
E4	false	One Flag	C5	GTFB	UNKNOWN	SYBR-None	24.151	24.090153
F3	false	No Flag	C6	GTFB	UNKNOWN	SYBR-None	23.587	23.57465
F4	false	No Flag	C6	GTFB	UNKNOWN	SYBR-None	23.562	23.57465
A5	false	No Flag	T1	GTFB	UNKNOWN	SYBR-None	23.449	23.722252
A6	false	One Flag	T1	GTFB	UNKNOWN	SYBR-None	23.996	23.722252
B5	false	No Flag	T2	GTFB	UNKNOWN	SYBR-None	23.108	23.177795
B6	false	No Flag	T2	GTFB	UNKNOWN	SYBR-None	23.247	23.177795
C5	true	Omitted by	T3	GTFB	UNKNOWN	SYBR-None		
C6	true	Omitted by	T3	GTFB	UNKNOWN	SYBR-None		
D5	false	One Flag	T5	GTFB	UNKNOWN	SYBR-None	23.35	23.30233
D6	false	No Flag	T5	GTFB	UNKNOWN	SYBR-None	23.255	23.30233
E5	true	Omitted by	T6	GTFB	UNKNOWN	SYBR-None		
E6	true	Omitted by	T6	GTFB	UNKNOWN	SYBR-None		

NaN		
NaN		
0.0231106	300	
0.0231106	300	
0.0215381	30	
0.0215381	30	
0.3024579	3	
0.3024579	3	
0.4138888	0.3	
0.4138888	0.3	
0.1622013	0.03	
0.1622013	0.03	
0.3290529	0.189691	0.1693288
0.3290529	0.1489666	0.1693288
0.1520159	0.1980378	0.1875773
0.1520159	0.1771168	0.1875773
0.0617367	0.1501844	0.1536676
0.0617367	0.1571509	0.1536676
0.3671321	0.0939918	0.1085368
0.3671321	0.1230818	0.1085368
0.086105	0.1033115	0.100146
0.086105	0.0969804	0.100146
0.0179026	0.1299663	0.1308264
0.0179026	0.1316865	0.1308264
0.3867327	0.1396603	0.122394
0.3867327	0.1051277	0.122394
0.0979992	0.1666571	0.1608702
0.0979992	0.1550833	0.1608702
0.0669103	0.14704	0.1507432
0.0669103	0.1544464	0.1507432

		Grupos	
		C	T
	1	0.17	0.12
	2	0.19	0.16
	3	0.15	
	4	0.11	
	5	0.10	0.15
	6	0.13	
Media		0.14	0.14
DP		0.03	0.02
	1	25.42	26.29
	2	25.84	25.45
	3	25.92	25.84
	4	26.07	
	5	26.40	25.34
	6	26.34	26.50
Media		26.00	25.88
DP		0.36	0.51
Normalizado			
	1	4.30	3.22
	2	4.85	4.09
	3	3.98	
	4	2.83	
	5	2.64	3.82
	6	3.45	
Media		3.68	3.71
DP		0.86	0.45
