# Supplementary material for: Trans,trans-farnesol, an antimicrobial natural compound, improves glass ionomer cement properties
Source: PLoS One. 2019 Aug 20;14(8):e0220718. doi: 10.1371/journal.pone.0220718 (PMC6701760; doi:10.1371/journal.pone.0220718)
Supplement: S4 Text — (PDF) [file pone.0220718.s008.pdf]

F1	false	No Flag		GTFC	NTC	SYBR-None	Undetermin	
F2	false	No Flag		GTFC	NTC	SYBR-None	Undetermin	
A1	true	Omitted by		GTFC	STANDARD	SYBR-None		
A2	true	Omitted by		GTFC	STANDARD	SYBR-None		
B1	false	No Flag		GTFC	STANDARD	SYBR-None	12.263	12.564425
B2	false	No Flag		GTFC	STANDARD	SYBR-None	12.866	12.564425
C1	false	No Flag		GTFC	STANDARD	SYBR-None	16.629	16.826813
C2	false	No Flag		GTFC	STANDARD	SYBR-None	17.025	16.826813
D1	false	No Flag		GTFC	STANDARD	SYBR-None	21.564	21.633158
D2	false	No Flag		GTFC	STANDARD	SYBR-None	21.703	21.633158
E1	false	No Flag		GTFC	STANDARD	SYBR-None	25.853	25.931938
E2	false	No Flag		GTFC	STANDARD	SYBR-None	26.011	25.931938
A3	false	No Flag	C1	GTFC	UNKNOWN	SYBR-None	22.276	22.400505
A4	false	No Flag	C1	GTFC	UNKNOWN	SYBR-None	22.525	22.400505
B3	false	No Flag	C2	GTFC	UNKNOWN	SYBR-None	22.144	22.310957
B4	false	No Flag	C2	GTFC	UNKNOWN	SYBR-None	22.478	22.310957
C3	false	No Flag	C3	GTFC	UNKNOWN	SYBR-None	21.833	21.864363
C4	false	No Flag	C3	GTFC	UNKNOWN	SYBR-None	21.896	21.864363
D3	false	No Flag	C4	GTFC	UNKNOWN	SYBR-None	21.9	22.008038
D4	false	No Flag	C4	GTFC	UNKNOWN	SYBR-None	22.116	22.008038
E3	false	No Flag	C5	GTFC	UNKNOWN	SYBR-None	22.72	22.727089
E4	false	No Flag	C5	GTFC	UNKNOWN	SYBR-None	22.734	22.727089
F3	false	No Flag	C6	GTFC	UNKNOWN	SYBR-None	22.104	22.178043
F4	false	No Flag	C6	GTFC	UNKNOWN	SYBR-None	22.252	22.178043
A5	false	No Flag	T1	GTFC	UNKNOWN	SYBR-None	22.196	22.26374
A6	false	No Flag	T1	GTFC	UNKNOWN	SYBR-None	22.331	22.26374
B5	false	No Flag	T2	GTFC	UNKNOWN	SYBR-None	22.301	22.324924
B6	false	No Flag	T2	GTFC	UNKNOWN	SYBR-None	22.349	22.324924
C5	true	Omitted by	T3	GTFC	UNKNOWN	SYBR-None		
C6	false	No Flag	T3	GTFC	UNKNOWN	SYBR-None	24.131	24.13116
D5	false	No Flag	T5	GTFC	UNKNOWN	SYBR-None	22.561	22.54253
D6	false	No Flag	T5	GTFC	UNKNOWN	SYBR-None	22.524	22.54253
E5	true	Omitted by	T6	GTFC	UNKNOWN	SYBR-None		
E6	false	No Flag	T6	GTFC	UNKNOWN	SYBR-None	26.534	26.533945

NaN		
NaN		
	300	
	300	
0.4268383	30	
0.4268383	30	
0.2800156	3	
0.2800156	3	
0.0985009	0.3	
0.0985009	0.3	
0.1117869	0.03	
0.1117869	0.03	
0.1758043	0.1999019	0.187939
0.1758043	0.1759761	0.187939
0.2364243	0.2139449	0.197092
0.2364243	0.180239	0.197092
0.0449279	0.2509551	0.2469332
0.0449279	0.2429113	0.2469332
0.1531488	0.2424615	0.2297194
0.1531488	0.2169773	0.2297194
0.0102757	0.159232	0.158641
0.0102757	0.15805	0.158641
0.104423	0.2183304	0.2103697
0.104423	0.2024091	0.2103697
0.0957185	0.2082865	0.2013034
0.0957185	0.1943203	0.2013034
0.0335651	0.1973561	0.1949834
0.0335651	0.1926107	0.1949834
NaN	0.0772248	0.0772248
0.026622	0.1727099	0.1743931
0.026622	0.1760763	0.1743931
NaN	0.0225274	0.0225274

	Grupos		
		C	T
	1	0.19	0.20
	2	0.20	0.19
	3	0.25	
	4	0.23	
	5	0.16	0.17
	6	0.21	
Media		0.21	0.19
DP		0.03	0.01
	1	25.42	26.29
	2	25.84	25.45
	3	25.92	25.84
	4	26.07	
	5	26.40	25.34
	6	26.34	26.50
Media		26.00	25.88
DP		0.36	0.51
Normalizado			
	1	4.78	5.29
	2	5.09	4.96
	3	6.40	
	4	5.99	
	5	4.19	4.42
	6	5.54	
Media		5.33	4.89
DP		0.81	0.44
