# Supplementary material for: Trans,trans-farnesol, an antimicrobial natural compound, improves glass ionomer cement properties
Source: PLoS One. 2019 Aug 20;14(8):e0220718. doi: 10.1371/journal.pone.0220718 (PMC6701760; doi:10.1371/journal.pone.0220718)
Supplement: S5 Text — (PDF) [file pone.0220718.s009.pdf]

F1	false	One Flag		GTFD	NTC	SYBR-None	28.902	
F2	false	One Flag		GTFD	NTC	SYBR-None	28.723	
A1	true	Omitted by		GTFD	STANDARD	SYBR-None		
A2	true	Omitted by		GTFD	STANDARD	SYBR-None		
B1	false	No Flag		GTFD	STANDARD	SYBR-None	10.324	10.30627
B2	false	No Flag		GTFD	STANDARD	SYBR-None	10.288	10.30627
C1	false	No Flag		GTFD	STANDARD	SYBR-None	14.728	14.800196
C2	false	No Flag		GTFD	STANDARD	SYBR-None	14.872	14.800196
D1	false	No Flag		GTFD	STANDARD	SYBR-None	20.171	20.284557
D2	false	No Flag		GTFD	STANDARD	SYBR-None	20.398	20.284557
E1	false	One Flag		GTFD	STANDARD	SYBR-None	24.804	24.674484
E2	false	One Flag		GTFD	STANDARD	SYBR-None	24.545	24.674484
A3	false	No Flag	C1	GTFD	UNKNOWN	SYBR-None	20.724	20.736912
A4	false	One Flag	C1	GTFD	UNKNOWN	SYBR-None	20.75	20.736912
B3	false	One Flag	C2	GTFD	UNKNOWN	SYBR-None	20.665	20.772182
B4	false	One Flag	C2	GTFD	UNKNOWN	SYBR-None	20.88	20.772182
C3	false	One Flag	C3	GTFD	UNKNOWN	SYBR-None	20.602	20.70138
C4	false	One Flag	C3	GTFD	UNKNOWN	SYBR-None	20.801	20.70138
D3	false	One Flag	C4	GTFD	UNKNOWN	SYBR-None	20.667	20.625233
D4	false	One Flag	C4	GTFD	UNKNOWN	SYBR-None	20.583	20.625233
E3	false	One Flag	C5	GTFD	UNKNOWN	SYBR-None	20.831	20.897509
E4	false	One Flag	C5	GTFD	UNKNOWN	SYBR-None	20.964	20.897509
F3	false	One Flag	C6	GTFD	UNKNOWN	SYBR-None	21.159	20.950481
F4	false	One Flag	C6	GTFD	UNKNOWN	SYBR-None	20.742	20.950481
A5	false	No Flag	T1	GTFD	UNKNOWN	SYBR-None	19.869	20.14573
A6	false	One Flag	T1	GTFD	UNKNOWN	SYBR-None	20.423	20.14573
B5	false	One Flag	T2	GTFD	UNKNOWN	SYBR-None	20.893	20.820452
B6	false	One Flag	T2	GTFD	UNKNOWN	SYBR-None	20.748	20.820452
C5	true	Omitted by	T3	GTFD	UNKNOWN	SYBR-None		
C6	false	One Flag	T3	GTFD	UNKNOWN	SYBR-None	22.853	22.853374
D5	false	One Flag	T5	GTFD	UNKNOWN	SYBR-None	20.69	20.720333
D6	false	One Flag	T5	GTFD	UNKNOWN	SYBR-None	20.75	20.720333
E5	true	Omitted by	T6	GTFD	UNKNOWN	SYBR-None		
E6	false	One Flag	T6	GTFD	UNKNOWN	SYBR-None	25.678	25.678324

NaN		
NaN		
	300	
	300	
0.0255713	30	
0.0255713	30	
0.1016346	3	
0.1016346	3	
0.1609983	0.3	
0.1609983	0.3	
0.1836483	0.03	
0.1836483	0.03	
0.0187523	0.2075087	0.206213
0.0187523	0.2049172	0.206213
0.1518352	0.2133757	0.2030535
0.1518352	0.1927313	0.2030535
0.1408312	0.2198442	0.209944
0.1408312	0.2000439	0.209944
0.0594574	0.2131272	0.2174592
0.0594574	0.2217912	0.2174592
0.0945357	0.1972481	0.1911935
0.0945357	0.1851389	0.1911935
0.2944534	0.1688503	0.1872681
0.2944534	0.2056858	0.1872681
0.3915881	0.3111461	0.2752361
0.3915881	0.2393262	0.2752361
0.1023258	0.1915239	0.1983213
0.1023258	0.2051188	0.1983213
NaN	0.075635	0.075635
0.0422426	0.2107983	0.2078563
0.0422426	0.2049142	0.2078563
NaN	0.0198301	0.0198301

		Grupos	
		C	T
	1	0.21	0.28
	2	0.20	0.20
	3	0.21	
	4	0.22	
	5	0.19	0.21
	6	0.19	
Media		0.20	0.23
DP		0.01	0.04
	1	25.42	26.29
	2	25.84	25.45
	3	25.92	25.84
	4	26.07	
	5	26.40	25.34
	6	26.34	26.50
Media		26.00	25.88
DP		0.36	0.51
Normalizado			
	1	5.24	7.24
	2	5.25	5.05
	3	5.44	
	4	5.67	
	5	5.05	5.27
	6	4.93	
Media		5.26	5.85
DP		0.27	1.21
