# Supplementary material for: Trans,trans-farnesol, an antimicrobial natural compound, improves glass ionomer cement properties
Source: PLoS One. 2019 Aug 20;14(8):e0220718. doi: 10.1371/journal.pone.0220718 (PMC6701760; doi:10.1371/journal.pone.0220718)
Supplement: S6 Text — (PDF) [file pone.0220718.s010.pdf]

F1	false	2 Flags		GBPB	NTC	SYBR-None	29.361	
F2	false	2 Flags		GBPB	NTC	SYBR-None	30.804	
A1	false	No Flag		GBPB	STANDARD	SYBR-None	7.906	7.8549795
A2	false	No Flag		GBPB	STANDARD	SYBR-None	7.804	7.8549795
B1	false	No Flag		GBPB	STANDARD	SYBR-None	11.281	11.363545
B2	false	No Flag		GBPB	STANDARD	SYBR-None	11.446	11.363545
C1	false	No Flag		GBPB	STANDARD	SYBR-None	16.714	16.833729
C2	false	No Flag		GBPB	STANDARD	SYBR-None	16.954	16.833729
D1	false	No Flag		GBPB	STANDARD	SYBR-None	22.502	22.399588
D2	false	No Flag		GBPB	STANDARD	SYBR-None	22.297	22.399588
E1	false	No Flag		GBPB	STANDARD	SYBR-None	26.885	26.775402
E2	false	No Flag		GBPB	STANDARD	SYBR-None	26.666	26.775402
A3	false	No Flag	C1	GBPB	UNKNOWN	SYBR-None	21.681	21.783325
A4	false	No Flag	C1	GBPB	UNKNOWN	SYBR-None	21.885	21.783325
B3	false	No Flag	C2	GBPB	UNKNOWN	SYBR-None	21.81	21.838104
B4	false	No Flag	C2	GBPB	UNKNOWN	SYBR-None	21.866	21.838104
C3	false	No Flag	C3	GBPB	UNKNOWN	SYBR-None	21.617	21.655258
C4	false	No Flag	C3	GBPB	UNKNOWN	SYBR-None	21.694	21.655258
D3	false	No Flag	C4	GBPB	UNKNOWN	SYBR-None	21.647	21.88365
D4	false	No Flag	C4	GBPB	UNKNOWN	SYBR-None	22.12	21.88365
E3	false	One Flag	C5	GBPB	UNKNOWN	SYBR-None	22.655	22.64981
E4	false	No Flag	C5	GBPB	UNKNOWN	SYBR-None	22.645	22.64981
F3	false	One Flag	C6	GBPB	UNKNOWN	SYBR-None	21.747	21.856367
F4	false	No Flag	C6	GBPB	UNKNOWN	SYBR-None	21.966	21.856367
A5	false	No Flag	T1	GBPB	UNKNOWN	SYBR-None	21.953	22.249096
A6	false	No Flag	T1	GBPB	UNKNOWN	SYBR-None	22.545	22.249096
B5	false	No Flag	T2	GBPB	UNKNOWN	SYBR-None	21.885	21.911808
B6	false	No Flag	T2	GBPB	UNKNOWN	SYBR-None	21.938	21.911808
C5	false	2 Flags	T3	GBPB	UNKNOWN	SYBR-None	25.449	24.47228
C6	false	One Flag	T3	GBPB	UNKNOWN	SYBR-None	23.496	24.47228
D5	false	No Flag	T5	GBPB	UNKNOWN	SYBR-None	22.31	22.332825
D6	false	No Flag	T5	GBPB	UNKNOWN	SYBR-None	22.355	22.332825
E5	false	2 Flags	T6	GBPB	UNKNOWN	SYBR-None	29.153	26.777754
E6	false	2 Flags	T6	GBPB	UNKNOWN	SYBR-None	24.403	26.777754

NaN		
NaN		
0.0727613	300	
0.0727613	300	
0.1161311	30	
0.1161311	30	
0.1697513	3	
0.1697513	3	
0.1448233	0.3	
0.1448233	0.3	
0.1549763	0.03	
0.1549763	0.03	
0.1442124	0.3377635	0.3222923
0.1442124	0.3068211	0.3222923
0.0392485	0.3178482	0.3137464
0.0392485	0.3096445	0.3137464
0.0542056	0.3481717	0.3419969
0.0542056	0.3358221	0.3419969
0.3340323	0.343201	0.3089626
0.3340323	0.2747243	0.3089626
0.0066855	0.2135501	0.2140267
0.0066855	0.2145034	0.2140267
0.1552299	0.3275386	0.3114478
0.1552299	0.2953569	0.3114478
0.4190557	0.2972208	0.2610183
0.4190557	0.2248157	0.2610183
0.0374278	0.3068148	0.3030367
0.0374278	0.2992586	0.3030367
1.3806957	0.0572585	0.1004601
1.3806957	0.1436616	0.1004601
0.0320518	0.2511648	0.2485115
0.0320518	0.2458582	0.2485115
3.3587193	0.0099994	0.0518573
3.3587193	0.0937152	0.0518573

	Grupos		
		C	T
	1	0.32	0.26
	2	0.31	0.30
	3	0.34	
	4	0.31	
	5	0.21	0.25
	6	0.31	
Media		0.30	0.27
DP		0.04	0.03
	1	25.42	26.29
	2	25.84	25.45
	3	25.92	25.84
	4	26.07	
	5	26.40	25.34
	6	26.34	26.50
Media		26.00	25.88
DP		0.36	0.51
Normalizado			
	1	8.19	6.86
	2	8.11	7.71
	3	8.87	
	4	8.05	
	5	5.65	6.30
	6	8.20	
Media		7.85	6.96
DP		1.12	0.71
