# Supplementary material for: Trans,trans-farnesol, an antimicrobial natural compound, improves glass ionomer cement properties
Source: PLoS One. 2019 Aug 20;14(8):e0220718. doi: 10.1371/journal.pone.0220718 (PMC6701760; doi:10.1371/journal.pone.0220718)
Supplement: S7 Text — (PDF) [file pone.0220718.s011.pdf]

F1	false	One Flag		vicR	NTC	SYBR-Non	29.536	
F2	false	One Flag		vicR	NTC	SYBR-Non	28.992	
A1	true	Omitted by		vicR	STANDARI	SYBR-None		
A2	true	Omitted by		vicR	STANDARI	SYBR-None		
B1	false	No Flag		vicR	STANDARI	SYBR-Non	11.597	11.610512
B2	false	No Flag		vicR	STANDARI	SYBR-Non	11.624	11.610512
C1	false	No Flag		vicR	STANDARI	SYBR-Non	16.255	16.326
C2	false	No Flag		vicR	STANDARI	SYBR-Non	16.397	16.326
D1	false	No Flag		vicR	STANDARI	SYBR-Non	21.44	21.40821
D2	false	No Flag		vicR	STANDARI	SYBR-Non	21.377	21.40821
E1	false	No Flag		vicR	STANDARI	SYBR-Non	25.636	25.804249
E2	false	One Flag		vicR	STANDARI	SYBR-Non	25.973	25.804249
A3	false	No Flag	C1	vicR	UNKNOWN	SYBR-Non	22.287	22.40038
A4	false	No Flag	C1	vicR	UNKNOWN	SYBR-Non	22.514	22.40038
B3	false	No Flag	C2	vicR	UNKNOWN	SYBR-Non	22.914	22.579906
B4	false	No Flag	C2	vicR	UNKNOWN	SYBR-Non	22.246	22.579906
C3	false	No Flag	C3	vicR	UNKNOWN	SYBR-Non	22.171	22.350243
C4	false	No Flag	C3	vicR	UNKNOWN	SYBR-Non	22.53	22.350243
D3	false	No Flag	C4	vicR	UNKNOWN	SYBR-Non	22.226	22.384964
D4	false	No Flag	C4	vicR	UNKNOWN	SYBR-Non	22.544	22.384964
E3	false	No Flag	C5	vicR	UNKNOWN	SYBR-Non	22.868	22.948214
E4	false	No Flag	C5	vicR	UNKNOWN	SYBR-Non	23.028	22.948214
F3	false	No Flag	C6	vicR	UNKNOWN	SYBR-Non	22.258	22.269835
F4	false	No Flag	C6	vicR	UNKNOWN	SYBR-Non	22.282	22.269835
A5	false	No Flag	T1	vicR	UNKNOWN	SYBR-Non	22.446	22.461197
A6	false	No Flag	T1	vicR	UNKNOWN	SYBR-Non	22.476	22.461197
B5	false	No Flag	T2	vicR	UNKNOWN	SYBR-Non	22.246	22.243038
B6	false	No Flag	T2	vicR	UNKNOWN	SYBR-Non	22.24	22.243038
C5	true	Omitted by	T3	vicR	UNKNOWN	SYBR-None		
C6	false	One Flag	T3	vicR	UNKNOWN	SYBR-Non	24.391	24.39092
D5	false	No Flag	T5	vicR	UNKNOWN	SYBR-Non	22.344	22.418259
D6	false	No Flag	T5	vicR	UNKNOWN	SYBR-Non	22.493	22.418259
E5	true	Omitted by	T6	vicR	UNKNOWN	SYBR-None		
E6	true	Omitted by	T6	vicR	UNKNOWN	SYBR-None		

NaN		
NaN		
	300	
	300	
0.01943	30	
0.01943	30	
0.100671	3	
0.100671	3	
0.0445947	0.3	
0.0445947	0.3	
0.2382207	0.03	
0.2382207	0.03	
0.1606651	0.1749354	0.1658425
0.1606651	0.1567497	0.1658425
0.4722969	0.1292137	0.153817
0.4722969	0.1784203	0.153817
0.2539452	0.185027	0.1702915
0.2539452	0.155556	0.1702915
0.2244546	0.1801257	0.1673216
0.2244546	0.1545175	0.1673216
0.1131127	0.1320943	0.1271825
0.1131127	0.1222706	0.1271825
0.0167535	0.177385	0.1763756
0.0167535	0.1753662	0.1763756
0.0210384	0.1619585	0.1608028
0.0210384	0.1596472	0.1608028
0.0037831	0.17844	0.1786709
0.0037831	0.1789018	0.1786709
NaN	0.0633015	0.0633015
0.1057124	0.1702057	0.1642761
0.1057124	0.1583464	0.1642761

	Grupos		
		C	T
	1	0.17	0.16
	2	0.15	0.18
	3	0.17	
	4	0.17	
	5	0.13	0.16
	6	0.18	
Media		0.16	0.17
DP		0.02	0.01
	1	25.42	26.29
	2	25.84	25.45
	3	25.92	25.84
	4	26.07	
	5	26.40	25.34
	6	26.34	26.50
Media		26.00	25.88
DP		0.36	0.51
Normalizado			
	1	4.22	4.23
	2	3.98	4.55
	3	4.41	
	4	4.36	
	5	3.36	4.16
	6	4.65	
Media		4.16	4.31
DP		0.45	0.21
