# Supplementary material for: Trans,trans-farnesol, an antimicrobial natural compound, improves glass ionomer cement properties
Source: PLoS One. 2019 Aug 20;14(8):e0220718. doi: 10.1371/journal.pone.0220718 (PMC6701760; doi:10.1371/journal.pone.0220718)
Supplement: S8 Text — (PDF) [file pone.0220718.s012.pdf]

Model Information	
Data Set	D.DADOS_AC_MTT
Response Variable	mtt
Response Distribution	Gaussian
Link Function	Identity
Variance Function	Default
Variance Matrix	Diagonal
Estimation Technique	Restricted Maximum Likelihood
Degrees of Freedom Method	Residual

Class Level Information		
Class	*Levels*	*Values*
trat	3	CIV CIV + tt-farnesol Control
bloco	3	1 2 3

Number of Observations Read	9
Number of Observations Used	9

Dimensions	
Covariance Parameters	1
Columns in X	7
Columns in Z	0
Subjects (Blocks in V)	1
Max Obs per Subject	9

Optimization Information	
Optimization Technique	None
Parameters	6
Lower Boundaries	1
Upper Boundaries	0
Fixed Effects	Not Profiled

Fit Statistics	
-2 Res Log Likelihood	28.41
AIC (smaller is better)	40.41
AICC (smaller is better)	124.41
BIC (smaller is better)	36.73

CAIC (smaller is better)	42.73
HQIC (smaller is better)	32.33
Pearson Chi-Square	94.90
Pearson Chi-Square / DF	23.73

Type III Tests of Fixed Effects				
Effect	*Num DF*	*Den DF*	*F Value*	*Pr > F*
bloco	2	4	7.83	0.0414
trat	2	4	52.81	0.0013

trat Least Squares Means					
Tratamento	*Estimate*	*Standard Error*	*DF*	*t Value*	*Pr > |t|*
CIV	60.6667	2.8122	4	21.57	<.0001
CIV + tt-farnesol	89.9500	2.8122	4	31.99	<.0001
Control	100.00	2.8122	4	35.56	<.0001

Differences of trat Least Squares Means<br>*Adjustment for Multiple Comparisons: Tukey*							
Tratamento	*Tratamento*	*Estimate*	*Standard Error*	*DF*	*t Value*	*Pr > |t|*	*Adj P*
CIV	CIV + tt-farnesol	-29.2833	3.9771	4	-7.36	0.0018	0.0040
CIV	Control	-39.3367	3.9771	4	-9.89	0.0006	0.0013
CIV + tt-farnesol	Control	-10.0533	3.9771	4	-2.53	0.0648	0.1323

Tukey Grouping for trat Least Squares Means (Alpha=0.05)		
LS-means with the same letter are not significantly different.		
Tratamento	*Estimate*	
Control	100.00	A
		A
CIV + tt-farnesol	89.9500	A
CIV	60.6667	B

Coeficiente de assimetria	*Coeficiente de curtose*	*W Shapiro-Wilk*	*Valor-p Ho:Normal*
0.060018	-0.45495	0.94873	0.67614

Analysis Variable : mtt Toxicidade (MTT)					
Tratamento	N Obs	Mean	Std Dev	Upper 95% CL for Mean	Lower 95% CL for Mean
CIV	3	60.67	10.43	86.59	34.75
CIV + tt-farnesol	3	89.95	7.26	107.98	71.92
Control	3	100.00	8.47	121.04	78.97

Obs	Tratamento	Bloco	Toxicidade (MTT)
1	Control	1	91.76
2	Control	2	99.57
3	Control	3	108.68
4	CIV + tt-farnesol	1	81.78
5	CIV + tt-farnesol	2	95.66
6	CIV + tt-farnesol	3	92.41
7	CIV	1	49.89
8	CIV	2	70.72
9	CIV	3	61.39
