# Supplementary material for: Trans,trans-farnesol, an antimicrobial natural compound, improves glass ionomer cement properties
Source: PLoS One. 2019 Aug 20;14(8):e0220718. doi: 10.1371/journal.pone.0220718 (PMC6701760; doi:10.1371/journal.pone.0220718)
Supplement: S9 Text — (PDF) [file pone.0220718.s013.pdf]

Model Information	
Data Set	D.DADOS_AC_MEC
Response Variable	td
Response Distribution	Gaussian
Link Function	Identity
Variance Function	Default
Variance Matrix	Diagonal
Estimation Technique	Restricted Maximum Likelihood
Degrees of Freedom Method	Residual

Class Level Information		
Class	*Levels*	*Values*
material	2	CIV TT MIC

Number of Observations Read	23
Number of Observations Used	14

Dimensions	
Covariance Parameters	1
Columns in X	3
Columns in Z	0
Subjects (Blocks in V)	1
Max Obs per Subject	14

Optimization Information	
Optimization Technique	None
Parameters	3
Lower Boundaries	1
Upper Boundaries	0
Fixed Effects	Not Profiled

Fit Statistics	
-2 Res Log Likelihood	73.56
AIC (smaller is better)	79.56
AICC (smaller is better)	82.56
BIC (smaller is better)	81.01
CAIC (smaller is better)	84.01

HQIC (smaller is better)	79.02
Pearson Chi-Square	233.75
Pearson Chi-Square / DF	19.48

Type III Tests of Fixed Effects				
Effect	*Num DF*	*Den DF*	*F Value*	*Pr > F*
material	1	12	2.76	0.1226

material Least Squares Means					
Material	*Estimate*	*Standard Error*	*DF*	*t Value*	*Pr > |t|*
CIV	17.8642	1.8018	12	9.91	<.0001
TT MIC	13.9050	1.5604	12	8.91	<.0001

Differences of material Least Squares Means Adjustment for Multiple Comparisons: Tukey-Kramer							
Material	*Material*	*Estimate*	*Standard Error*	*DF*	*t Value*	*Pr > |t|*	*Adj P*
CIV	TT MIC	3.9592	2.3836	12	1.66	0.1226	0.1226

Tukey-Kramer Grouping for material Least Squares Means (Alpha=0.05)		
LS-means with the same letter are not significantly different.		
Material	*Estimate*	
CIV	17.8642	A
		A
TT MIC	13.9050	A

Material	*Tração diametral*	*Resíduo*
CIV	7.315	-10.5492
TT MIC	8.550	-5.3550
TT MIC	11.100	-2.8050
TT MIC	12.010	-1.8950
TT MIC	12.930	-0.9750
CIV	19.720	1.8558
TT MIC	16.470	2.5650
TT MIC	16.590	2.6850
TT MIC	18.810	4.9050

CIV	23.800	5.9358

Coeficiente de assimetria	*Coeficiente de curtose*	*W Shapiro-Wilk*	*Valor-p Ho:Normal*
-1.15449	1.92001	0.92404	0.25124

Analysis Variable : td Tração diametral					
Material	*N Obs*	*Mean*	*Std Dev*	*Upper 95% CL for Mean*	*Lower 95% CL for Mean*
CIV	10	17.86	5.55	23.69	12.04
TT MIC	13	13.91	3.37	16.73	11.08

Model Information	
Data Set	D.DADOS_AC_MEC
Response Variable	rc
Response Distribution	Gaussian
Link Function	Identity
Variance Function	Default
Variance Matrix	Diagonal
Estimation Technique	Restricted Maximum Likelihood
Degrees of Freedom Method	Residual

Class Level Information		
Class	*Levels*	*Values*
material	2	CIV TT MIC

Number of Observations Read	23
Number of Observations Used	23

Dimensions	
Covariance Parameters	1
Columns in X	3
Columns in Z	0
Subjects (Blocks in V)	1
Max Obs per Subject	23

Optimization Information	
Optimization Technique	None
Parameters	3
Lower Boundaries	1
Upper Boundaries	0

Fixed Effects	Not Profiled

Fit Statistics	
-2 Res Log Likelihood	154.65
AIC (smaller is better)	160.65
AICC (smaller is better)	162.06
BIC (smaller is better)	163.78
CAIC (smaller is better)	166.78
HQIC (smaller is better)	161.33
Pearson Chi-Square	1539.03
Pearson Chi-Square / DF	73.29

Type III Tests of Fixed Effects

Effect	*Num DF*	*Den DF*	*F Value*	*Pr > F*
material	1	21	1.68	0.2090

material Least Squares Means

Material	*Estimate*	*Standard Error*	*DF*	*t Value*	*Pr > |t|*
CIV	23.8740	2.7072	21	8.82	<.0001
TT MIC	28.5408	2.3743	21	12.02	<.0001

Differences of material Least Squares Means
Adjustment for Multiple Comparisons: Tukey-Kramer

Material	*Material*	*Estimate*	*Standard Error*	*DF*	*t Value*	*Pr > |t|*	*Adj P*
CIV	TT MIC	-4.6668	3.6009	21	-1.30	0.2090	0.2090

Tukey-Kramer Grouping for material Least Squares Means (Alpha=0.05)

LS-means with the same letter are not significantly different.

Material	*Estimate*	
TT MIC	28.5408	A
		A
CIV	23.8740	A

Material	*Compressão (MPa)*	*Resíduo*
TT MIC	18.16	-10.3808

CIV	13.62	-10.2540
TT MIC	19.87	-8.6708
TT MIC	19.88	-8.6608
TT MIC	20.31	-8.2308
CIV	34.11	10.2360
TT MIC	38.89	10.3492
TT MIC	39.42	10.8792
TT MIC	42.83	14.2892
CIV	42.51	18.6360

Coeficiente de assimetria	*Coeficiente de curtose*	*W Shapiro-Wilk*	*Valor-p Ho:Normal*
0.70094	-0.46712	0.92662	0.092432

Analysis Variable : rc Compressão (MPa)					
Material	*N Obs*	*Mean*	*Std Dev*	*Upper 95% CL for Mean*	*Lower 95% CL for Mean*
CIV	10	23.87	8.94	30.27	17.48
TT MIC	13	28.54	8.27	33.54	23.55

Model Information	
Data Set	D.DADOS_AC_MEC
Response Variable	ra
Response Distribution	Gaussian
Link Function	Identity
Variance Function	Default
Variance Matrix	Diagonal
Estimation Technique	Restricted Maximum Likelihood
Degrees of Freedom Method	Residual

Class Level Information		
Class	*Levels*	*Values*
material	2	CIV TT MIC

Number of Observations Read	23
Number of Observations Used	19

Dimensions	
Covariance Parameters	1
Columns in X	3
Columns in Z	0

Subjects (Blocks in V)	1
Max Obs per Subject	19

Optimization Information	
Optimization Technique	None
Parameters	3
Lower Boundaries	1
Upper Boundaries	0
Fixed Effects	Not Profiled

Fit Statistics	
-2 Res Log Likelihood	-21.48
AIC (smaller is better)	-15.48
AICC (smaller is better)	-13.64
BIC (smaller is better)	-12.98
CAIC (smaller is better)	-9.98
HQIC (smaller is better)	-15.23
Pearson Chi-Square	0.22
Pearson Chi-Square / DF	0.01

Type III Tests of Fixed Effects

Effect	*Num DF*	*Den DF*	*F Value*	*Pr > F*
material	1	17	3.68	0.0721

material Least Squares Means

Material	*Estimate*	*Standard Error*	*DF*	*t Value*	*Pr > |t|*
CIV	0.6771	0.03756	17	18.02	<.0001
TT MIC	0.7764	0.03564	17	21.79	<.0001

Differences of material Least Squares Means
Adjustment for Multiple Comparisons: Tukey-Kramer

Material	*Material*	*Estimate*	*Standard Error*	*DF*	*t Value*	*Pr > |t|*	*Adj P*
CIV	TT MIC	-0.09930	0.05178	17	-1.92	0.0721	0.0721

Tukey-Kramer Grouping for material Least Squares Means (Alpha=0.05)

LS-means with the same letter are not significantly different.

Material	Estimate	
TT MIC	0.7764	A
		A
CIV	0.6771	A

Material	Rugosidade	Resíduo
TT MIC	0.6094	-0.16698
CIV	0.5128	-0.16428
TT MIC	0.6233	-0.15308
TT MIC	0.6500	-0.12638
CIV	0.5568	-0.12028
TT MIC	0.8757	0.09932
TT MIC	0.8859	0.10952
TT MIC	0.9002	0.12382
CIV	0.8200	0.14292
TT MIC	0.9390	0.16262

Coeficiente de assimetria	Coeficiente de curtose	W Shapiro-Wilk	Valor-p Ho:Normal
-0.21998	-1.28984	0.93119	0.18213

Analysis Variable : ra Rugosidade					
Material	N Obs	Mean	Std Dev	Upper 95% CL for Mean	Lower 95% CL for Mean
CIV	10	0.68	0.10	0.75	0.60
TT MIC	13	0.78	0.12	0.86	0.69

Model Information	
Data Set	D.DADOS_AC_MEC
Response Variable	khn
Response Distribution	Gaussian
Link Function	Identity
Variance Function	Default
Variance Matrix	Diagonal
Estimation Technique	Restricted Maximum Likelihood
Degrees of Freedom Method	Residual

Class Level Information		
Class	Levels	Values
material	2	CIV TT MIC

Number of Observations Read	23
Number of Observations Used	20

Dimensions	
Covariance Parameters	1
Columns in X	3
Columns in Z	0
Subjects (Blocks in V)	1
Max Obs per Subject	20

Optimization Information	
Optimization Technique	None
Parameters	3
Lower Boundaries	1
Upper Boundaries	0
Fixed Effects	Not Profiled

Fit Statistics	
-2 Res Log Likelihood	127.45
AIC (smaller is better)	133.45
AICC (smaller is better)	135.16
BIC (smaller is better)	136.12
CAIC (smaller is better)	139.12
HQIC (smaller is better)	133.81
Pearson Chi-Square	969.68
Pearson Chi-Square / DF	53.87

Type III Tests of Fixed Effects

Effect	*Num DF*	*Den DF*	*F Value*	*Pr > F*
material	1	18	73.92	<.0001

material Least Squares Means

Material	*Estimate*	*Standard Error*	*DF*	*t Value*	*Pr > |t|*
CIV	44.1610	2.3210	18	19.03	<.0001
TT MIC	72.3830	2.3210	18	31.19	<.0001

Differences of material Least Squares Means<br>Adjustment for Multiple Comparisons: Tukey							
Material	*Material*	*Estimate*	*Standard Error*	*DF*	*t Value*	*Pr > |t|*	*Adj P*
CIV	TT MIC	-28.2220	3.2824	18	-8.60	<.0001	<.0001

Tukey Grouping for material Least Squares Means (Alpha=0.05)		
LS-means with the same letter are not significantly different.		
Material	*Estimate*	
TT MIC	72.3830	A
CIV	44.1610	B

Material	*Dureza*	*Resíduo*
CIV	30.30	-13.861
TT MIC	61.03	-11.353
CIV	36.90	-7.261
TT MIC	67.10	-5.283
CIV	38.90	-5.261
TT MIC	77.50	5.117
CIV	51.11	6.949
CIV	53.40	9.239
TT MIC	84.20	11.817
CIV	56.20	12.039

Coeficiente de assimetria	*Coeficiente de curtose*	*W Shapiro-Wilk*	*Valor-p Ho:Normal*
-0.014129	-0.48652	0.97347	0.82576

Analysis Variable : khn Dureza					
Material	*N Obs*	*Mean*	*Std Dev*	*Upper 95% CL for Mean*	*Lower 95% CL for Mean*
CIV	10	44.16	8.05	49.92	38.40
TT MIC	13	72.38	6.55	77.07	67.70

Obs	*Material*	*Tração diametral*	*Compressão (MPa)*	*Rugosidade*	*Dureza*
1	CIV	19.500	18.48	0.8200	42.80
2	CIV	17.800	21.01	0.5128	30.30
3	CIV	19.720	34.11	0.6149	53.40
4	CIV	19.050	30.20	0.7404	36.90
5	CIV	23.800	13.62	0.5568	41.50

6	CIV	7.315	19.42	.	48.50
7	CIV	.	21.15	0.7078	56.20
8	CIV	.	42.51	0.6960	38.90
9	CIV	.	16.66	0.7650	42.00
10	CIV	.	21.58	0.6800	51.11
11	TT MIC	12.010	29.33	0.7037	69.40
12	TT MIC	16.590	20.31	0.7710	67.10
13	TT MIC	11.100	28.25	0.6233	70.60
14	TT MIC	14.780	19.87	0.9002	76.80
15	TT MIC	8.550	18.16	0.8757	68.00
16	TT MIC	18.810	19.88	0.8859	77.50
17	TT MIC	16.470	22.31	0.8056	84.20
18	TT MIC	12.930	33.03	0.6094	75.80
19	TT MIC	.	30.70	0.9390	73.40
20	TT MIC	.	42.83	0.6500	61.03
21	TT MIC	.	39.42	.	.
22	TT MIC	.	28.05	.	.
23	TT MIC	.	38.89	.	.
