# Supplementary material for: Trans,trans-farnesol, an antimicrobial natural compound, improves glass ionomer cement properties
Source: PLoS One. 2019 Aug 20;14(8):e0220718. doi: 10.1371/journal.pone.0220718 (PMC6701760; doi:10.1371/journal.pone.0220718)
Supplement: S10 Text — (PDF) [file pone.0220718.s014.pdf]

```
GET DATA /TYPE=XLSX
  /FILE='J:\NYU Dental\Simone Duarte\Aline Castilho\Table biofilm Castilho mj.xlsx'
  /SHEET=name 'exp1-4 ASP'
  /CELLRANGE=full
  /READNAMES=on
  /ASSUMEDSTRWIDTH=32767.
EXECUTE.
DATASET NAME DataSet5 WINDOW=FRONT.

SAVE OUTFILE='J:\NYU Dental\Simone Duarte\Aline Castilho\ASP exp 1-4.sav'
  /COMPRESSED.
GRAPH
  /LINE(MULTIPLE)=MEAN(ASP) BY Time BY Group
  /INTERVAL SE(1.0).
```

Graph

Notes

Output Created		
Comments		
Input	Data	
	Active Dataset	
	Filter	
	Weight	
	Split File	
	N of Rows in Working Data File	
Syntax		
Resources	Processor Time	
	Elapsed Time	

[DataSet5] J:\NYU Dental\Simone Duarte\Aline Castilho\ASP exp 1-4.sav

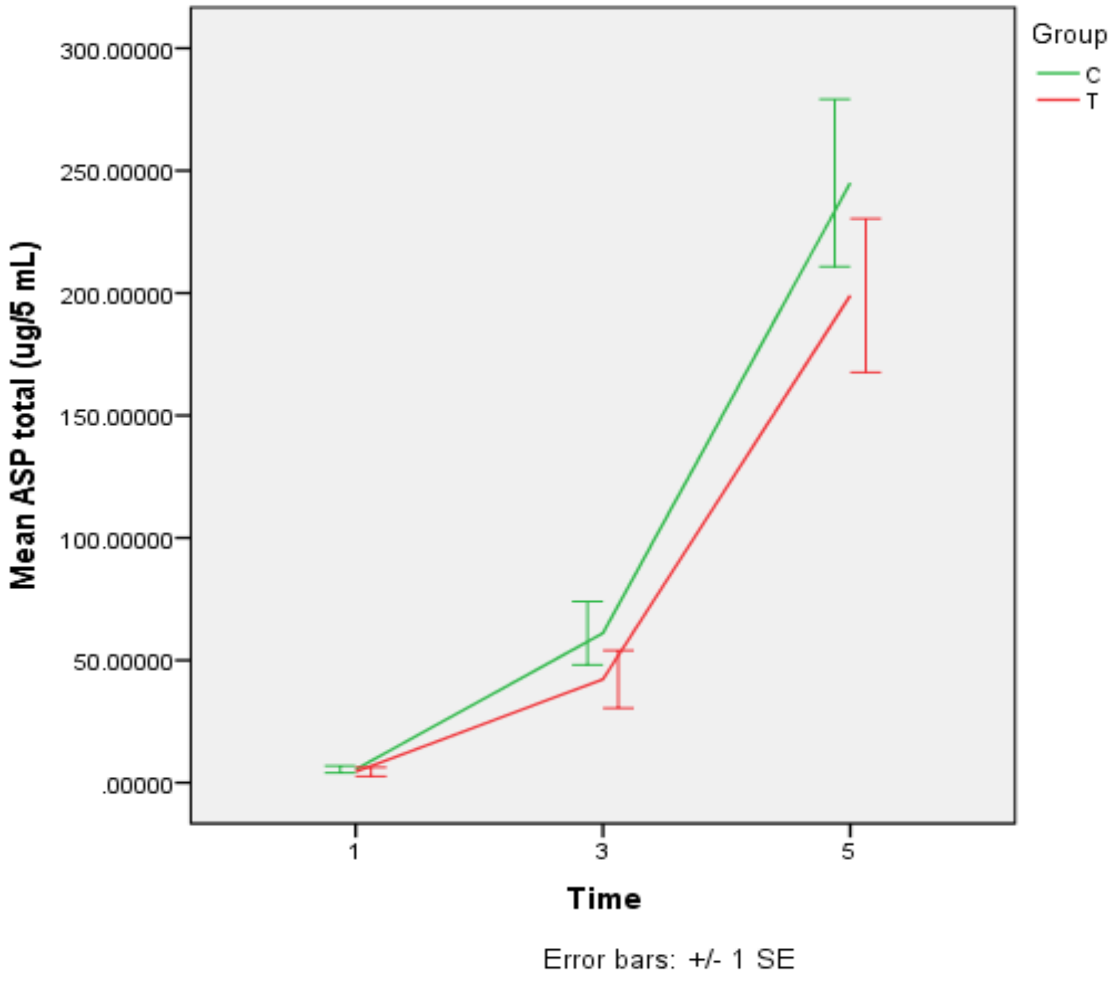


```
MIXED ASP BY Time Group
  /CRITERIA=CIN(95) MXITER(100) MXSTEP(10) SCORING(1) SINGULAR(0.000000000001) HCONVERGE(0
  /FIXED=Time Group Time*Group | SSTYPE(3)
  /METHOD=REML
  /PRINT=DESCRIPTIVES  SOLUTION TESTCOV
```

```
/RANDOM=INTERCEPT | SUBJECT(ID) COVTYPE(VC)
/EMMEANS=TABLES(Time)
/EMMEANS=TABLES(Group)
/EMMEANS=TABLES(Time*Group) .
```

Mixed Model Analysis

Notes

Output Created	
Comments	
Input	Data
	Active Dataset
	Filter
	Weight
	Split File
	N of Rows in Working Data File
Missing Value Handling	Definition of Missing
	Cases Used

Syntax	
Resources	Processor Time
	Elapsed Time

Descriptive Sta

ASP total (ug/5 mL)

ID	
1	1
2	1
3	1
4	1
5	3
6	3
7	3
8	3
9	5

10	5
11	5
12	5
13	1
14	1
15	1
16	1
17	3
18	3
19	3
20	3
21	5
22	5
23	5
24	5
25	1
26	1
27	1
28	1
29	3
30	3
31	3
32	3
33	5
34	5
35	5
36	5
37	1
38	1
39	1
40	1
41	3
42	3

43	3
44	3
45	5
46	5
47	5
48	5
Total	1
	3
	5
	Total

Totals that are aggregated over either a single category of a variable or a split file variable are omitted.

Model Dimension[a]

Fixed Effects	Intercept
	Time
	Group
	Time * Group
Random Effects	Intercept[b]
Residual	
Total	

a. Dependent Variable: ASP total (ug/5 mL).

b. As of version 11.5, the syntax rules for the RANDOM subcommand have changed. Your command syntax may yield results that differ
syntax reference guide for more information.

Information Criteria[a]

-2 Restricted Log Likelihood	1388.401
Akaike's Information Criterion (AIC)	1392.401
Hurvich and Tsai's Criterion (AICC)	1392.490
Bozdogan's Criterion (CAIC)	1400.255
Schwarz's Bayesian Criterion (BIC)	1398.255

The information criteria are displayed in smaller-is-better form.

a. Dependent Variable: ASP total (ug/5 mL).

Fixed Effects

Type III Tests of Fixed Effects[a]

Source	Numerator df
Intercept	1
Time	2
Group	1
Time * Group	2

a. Dependent Variable: ASP total (ug/5 mL).

Covariance Parameters

Estimates of

Parameter		
Residual		
Intercept [subject = ID]	Variance	

a. Dependent Variable: ASP total (ug/5 mL).

Estimated Marginal Means

1. Time[a]

Time	Mean
1	4.942
3	51.668
5	221.994

a. Dependent Variable: ASP total (ug/5 mL).

2. Group[a]

Group	Mean
C	103.839
T	81.897

a. Dependent Variable: ASP total (ug/5 mL).

3. Time * Gr

Time	
1	C
	T
3	C

	T
5	C
	T

a. Dependent Variable: ASP total (ug/5 mL).

```
MIXED RASP BY Time Group
  /CRITERIA=CIN(95) MXITER(100) MXSTEP(10) SCORING(1) SINGULAR(0.000000000001) HCONVERGE(0
  /FIXED=Time Group Time*Group | SSTYPE(3)
  /METHOD=REML
  /PRINT=DESCRIPTIVES  SOLUTION TESTCOV
  /RANDOM=INTERCEPT | SUBJECT(ID) COVTYPE(VC)
  /EMMEANS=TABLES(Time)
  /EMMEANS=TABLES(Group)
  /EMMEANS=TABLES(Time*Group) .
```

Mixed Model Analysis

Notes

Output Created	
Comments	
Input	Data
	Active Dataset
	Filter
	Weight
	Split File
	N of Rows in Working Data File
Missing Value Handling	Definition of Missing
	Cases Used

Syntax	
Resources	Processor Time
	Elapsed Time

Descriptive Sta

Rank of ASP	
ID	
1	1
2	1
3	1
4	1
5	3
6	3
7	3
8	3
9	5

10	5
11	5
12	5
13	1
14	1
15	1
16	1
17	3
18	3
19	3
20	3
21	5
22	5
23	5
24	5
25	1
26	1
27	1
28	1
29	3
30	3
31	3
32	3
33	5
34	5
35	5
36	5
37	1
38	1
39	1
40	1
41	3
42	3

43	3
44	3
45	5
46	5
47	5
48	5
Total	1
	3
	5
	Total

Totals that are aggregated over either a single category of a variable or a split file variable are omitted.

Model Dimension[a]

Fixed Effects	Intercept
	Time
	Group
	Time * Group
Random Effects	Intercept[b]
Residual	
Total	

a. Dependent Variable: Rank of ASP.

b. As of version 11.5, the syntax rules for the RANDOM subcommand have changed. Your command syntax may yield results that differ
syntax reference guide for more information.

Information Criteria[a]

-2 Restricted Log Likelihood	1045.994
Akaike's Information Criterion (AIC)	1049.994
Hurvich and Tsai's Criterion (AICC)	1050.083
Bozdogan's Criterion (CAIC)	1057.849
Schwarz's Bayesian Criterion (BIC)	1055.849

The information criteria are displayed in smaller-is-better form.

a. Dependent Variable: Rank of ASP.

Fixed Effects

Type III Tests of Fixed Effects[a]

Source	Numerator df
Intercept	1
Time	2
Group	1
Time * Group	2

a. Dependent Variable: Rank of ASP.

Covariance Parameters

Estimates of

Parameter	
Residual	
Intercept [subject = ID]	Variance

a. Dependent Variable: Rank of ASP.

Estimated Marginal Means

1. Time[a]

Time	Mean
1	49.875
3	65.854
5	101.771

a. Dependent Variable: Rank of ASP.

2. Group[a]

Group	Mean
C	75.444
T	69.556

a. Dependent Variable: Rank of ASP.

3. Time * Gr

Time	
1	C
	T
3	C
	T

5	C	
	T	

a. Dependent Variable: Rank of ASP.

```
GRAPH
  /LINE(MULTIPLE)=MEAN(RASP) BY Time BY Group
  /INTERVAL SE(1.0).
```

Graph

Notes

Output Created		
Comments		
Input	Data	
	Active Dataset	
	Filter	
	Weight	
	Split File	
	N of Rows in Working Data File	
Syntax		
Resources	Processor Time	
	Elapsed Time	

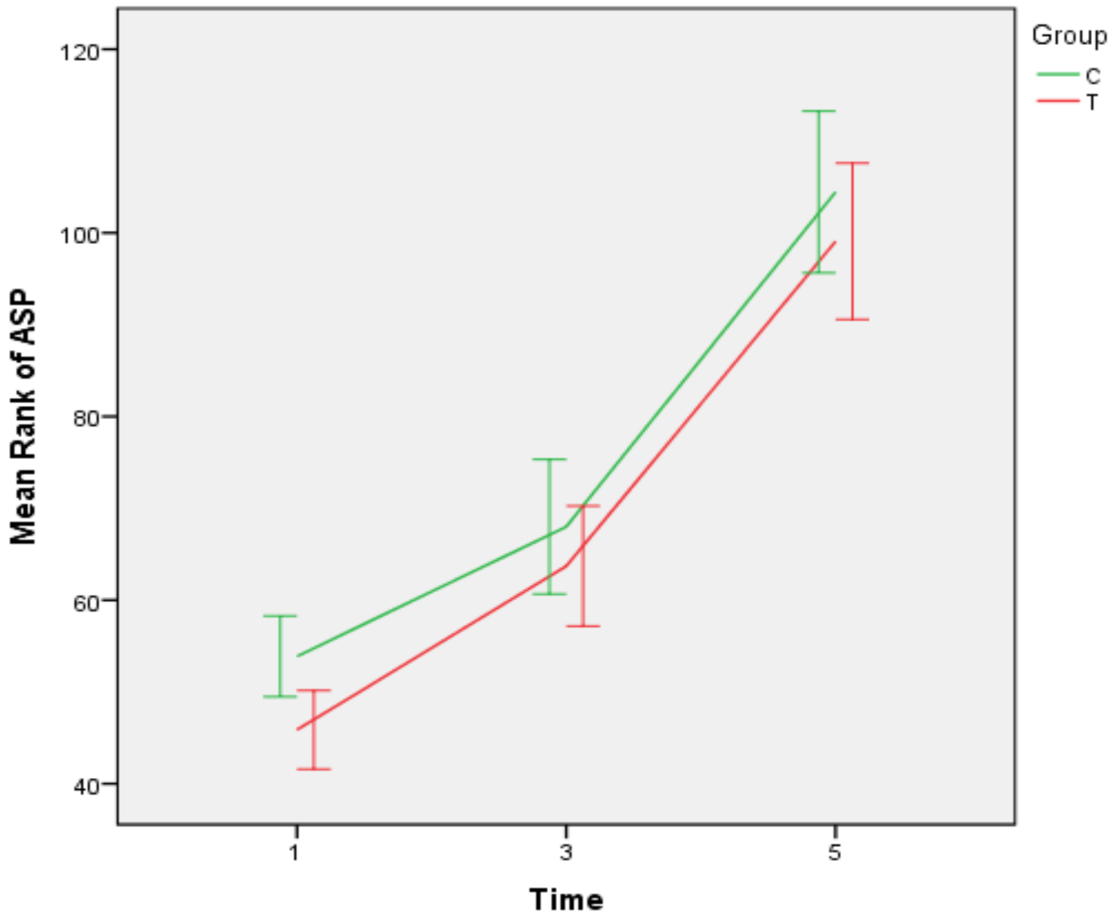

Error bars: +/- 1 SE

```
SORT CASES  BY Time.
SPLIT FILE SEPARATE BY Time.
MIXED RASP BY Group
  /CRITERIA=CIN(95) MXITER(100) MXSTEP(10) SCORING(1) SINGULAR(0.000000000001) HCONVERGE(0
  /FIXED=Group | SSTYPE(3)
  /METHOD=REML
  /PRINT=DESCRIPTIVES  SOLUTION TESTCOV
  /RANDOM=INTERCEPT | SUBJECT(ID) COVTYPE(VC)
  /EMMEANS=TABLES(Group) .
```

Mixed Model Analysis

Notes

Output Created		
Comments		
Input	Data	
	Active Dataset	
	Filter	
	Weight	
	Split File	
	N of Rows in Working Data File	
Missing Value Handling	Definition of Missing	
	Cases Used	
Syntax		
Resources	Processor Time	

	Elapsed Time

Warnings

For split file Time = ., no valid cases were found. Possible reasons are: all cases in this split file contain missing values in one or more variables, or all case weights (if specified) are less than 1/2, or all regression weights (if specified) are non-positive. No analyses will be performed for this split file.

Time = 1

Descriptive Statistics[a]

Rank of ASP

ID	
1	T
2	T
3	C
4	C
13	T
14	T
15	C
16	C
25	T
26	T
27	C
28	C
37	T
38	T
39	C
40	C
Total	C
	T

	Total

Totals that are aggregated over either a single category of a variable or a split file variable are omitted.

a. Time = 1

Model Dimension[a,b]

Fixed Effects	Intercept
	Group
Random Effects	Intercept[c]
Residual	
Total	

a. Time = 1

b. Dependent Variable: Rank of ASP.

c. As of version 11.5, the syntax rules for the RANDOM subcommand have changed. Your command syntax may yield results that differ
syntax reference guide for more information.

Information Criteria[a,b]

-2 Restricted Log Likelihood	354.513
Akaike's Information Criterion (AIC)	358.513
Hurvich and Tsai's Criterion (AICC)	358.792
Bozdogan's Criterion (CAIC)	364.170
Schwarz's Bayesian Criterion (BIC)	362.170

The information criteria are displayed in smaller-is-better form.

a. Time = 1

b. Dependent Variable: Rank of ASP.

Fixed Effects

Type III Tests of Fixed Effects[a,b]

Source	Numerator df
Intercept	1
Group	1

a. Time = 1

b. Dependent Variable: Rank of ASP.

Covariance Parameters

Estimates of (

Parameter	
Residual	
Intercept [subject = ID]	Variance

a. Time = 1

b. Dependent Variable: Rank of ASP.

Estimated Marginal Means

Group[a,b]

Group	Mean

C	53.875
T	45.875

a. Time = 1

b. Dependent Variable: Rank of ASP.

Time = 3

Descriptive Statistics[a]

Rank of ASP

ID	
5	T
6	T
7	C
8	C
17	T
18	T
19	C
20	C
29	T
30	T
31	C
32	C
41	T
42	T
43	C
44	C
Total	C
	T
	Total

Totals that are aggregated over either a single category of a variable or a split file variable are omitted.

a. Time = 3

Model Dimension[a,b]

Fixed Effects	Intercept
	Group
Random Effects	Intercept[c]
Residual	
Total	

a. Time = 3

b. Dependent Variable: Rank of ASP.

c. As of version 11.5, the syntax rules for the RANDOM subcommand have changed. Your command syntax may yield results that differ
syntax reference guide for more information.

Information Criteria[a,b]

-2 Restricted Log Likelihood	313.659
Akaike's Information Criterion (AIC)	317.659
Hurvich and Tsai's Criterion (AICC)	317.938
Bozdogan's Criterion (CAIC)	323.316
Schwarz's Bayesian Criterion (BIC)	321.316

The information criteria are displayed in smaller-is-better form.

a. Time = 3

b. Dependent Variable: Rank of ASP.

Fixed Effects

Type III Tests of Fixed Effects[a,b]

Source	Numerator df
Intercept	1
Group	1

a. Time = 3

b. Dependent Variable: Rank of ASP.

Covariance Parameters

Estimates of [illegible]

Parameter	
Residual	
Intercept [subject = ID]	Variance

a. Time = 3

b. Dependent Variable: Rank of ASP.

Estimated Marginal Means

Group[a,b]

Group	Mean
C	68.000
T	63.708

a. Time = 3

b. Dependent Variable: Rank of ASP.

Time = 5

Descriptive Statistics[a]

Rank of ASP

ID	
9	T
10	T
11	C
12	C
21	T
22	T
23	C
24	C
33	T
34	T
35	C
36	C
45	T
46	T
47	C
48	C
Total	C
	T
	Total

Totals that are aggregated over either a single category of a variable or a split file variable are omitted.

a. Time = 5

Model Dimension[a,b]

Fixed Effects	Intercept
	Group
Random Effects	Intercept[c]
Residual	
Total	

a. Time = 5

b. Dependent Variable: Rank of ASP.

c. As of version 11.5, the syntax rules for the RANDOM subcommand have changed. Your command syntax may yield results that differ
syntax reference guide for more information.

Information Criteria[a,b]

-2 Restricted Log Likelihood	349.502
Akaike's Information Criterion (AIC)	353.502
Hurvich and Tsai's Criterion (AICC)	353.781
Bozdogan's Criterion (CAIC)	359.159
Schwarz's Bayesian Criterion (BIC)	357.159

The information criteria are displayed in smaller-is-better form.

a. Time = 5

b. Dependent Variable: Rank of ASP.

Fixed Effects

Type III Tests of Fixed Effects[a,b]

Source	Numerator df
Intercept	1
Group	1

a. Time = 5

b. Dependent Variable: Rank of ASP.

Covariance Parameters

Estimates of [illegible]

Parameter	
Residual	
Intercept [subject = ID]	Variance

a. Time = 5

b. Dependent Variable: Rank of ASP.

Estimated Marginal Means

Group[a,b]

Group	Mean
C	104.458
T	99.083

a. Time = 5

b. Dependent Variable: Rank of ASP.

05-JUL-2016 15:14:03

J:\NYU Dental\Simone Duarte\Aline Castilho\ASP exp 1-4.sav

DataSet5

<none>

<none>

<none>

227

GRAPH /LINE(MULTIPLE)=MEAN(ASP) BY Time BY Group /INTERVAL SE(1.0).

00:00:00.11

00:00:00.10

```
), ABSOLUTE) LCONVERGE(0, ABSOLUTE) PCONVERGE(0.000001, ABSOLUTE)
```

05-JUL-2016 15:14:50

J:\NYU Dental\Simone Duarte\Aline Castilho\ASP exp 1-4.sav

DataSet5

<none>

<none>

<none>

227

User-defined missing values are treated as missing.

Statistics are based on all cases with valid data for all variables in the model.

MIXED ASP BY Time Group
/CRITERIA=CIN(95) MXITER(100)
MXSTEP(10) SCORING(1)
SINGULAR(0.000000000001)
HCONVERGE(0, ABSOLUTE)
LCONVERGE(0, ABSOLUTE)
PCONVERGE(0.000001, ABSOLUTE)
/FIXED=Time Group Time*Group |
SSTYPE(3) /METHOD=REML
/PRINT=DESCRIPTIVES SOLUTION
TESTCOV /RANDOM=INTERCEPT |
SUBJECT(ID) COVTYPE(VC)
/EMMEANS=TABLES(Time)
/EMMEANS=TABLES(Group)
/EMMEANS=TABLES(Time*Group) .

00:00:00.02

00:00:00.03

atistics

	Count	Mean	Standard Deviation	Coefficient of Variation
T	3	0.0000000	0.00000000	
T	3	0.0000000	0.00000000	
C	3	0.0000000	0.00000000	
C	3	0.0000000	0.00000000	
T	3	0.0000000	0.00000000	
T	3	0.0000000	0.00000000	
C	3	0.0000000	0.00000000	
C	3	0.0000000	0.00000000	
T	3	0.0000000	0.00000000	

T	3	0.0000000	0.00000000	
C	3	0.0000000	0.00000000	
C	3	0.0000000	0.00000000	
T	3	0.0000000	0.00000000	
T	3	0.0000000	0.00000000	
C	3	0.0000000	0.00000000	
C	3	0.0000000	0.00000000	
T	3	0.0000000	0.00000000	
T	3	0.0000000	0.00000000	
C	3	0.0000000	0.00000000	
C	3	0.0000000	0.00000000	
T	3	305.4769231	22.43344924	7.3%
T	3	299.5897436	31.26753551	10.4%
C	3	255.2564103	12.12846750	4.8%
C	3	213.0769231	12.44443270	5.8%
T	3	0.0000000	0.00000000	
T	3	26.7948718	.36933759	1.4%
C	3	9.4461538	3.14569303	33.3%
C	3	18.6153846	2.27671517	12.2%
T	3	75.9230769	3.05278997	4.0%
T	3	79.1384615	3.80756022	4.8%
C	3	143.2051282	3.59678796	2.5%
C	3	131.4871795	5.54575019	4.2%
T	3	278.7179487	7.27536710	2.6%
T	3	418.6205128	42.17933349	10.1%
C	3	301.6923077	13.67674137	4.5%
C	3	504.4923077	27.48124697	5.4%
T	3	5.5255590	4.13174330	74.8%
T	3	3.1296308	5.42067950	173.2%
C	3	6.4999846	.62569332	9.6%
C	3	9.0564205	5.18678180	57.3%
T	3	156.6416564	61.83154154	39.5%
T	3	26.3782615	11.20966147	42.5%

C	3	108.2184872	10.55309818	9.8%
C	3	105.6964103	5.55699623	5.3%
T	3	56.8834923	56.35118169	99.1%
T	3	232.7155692	31.16172541	13.4%
C	3	306.7031077	13.62736501	4.4%
C	3	378.6844564	22.33438836	5.9%
C	24	5.4522429	6.76111616	124.0%
T	24	4.4312577	9.08125762	204.9%
Total	48	4.9417503	7.93684294	160.6%
C	24	61.0759006	63.54874300	104.0%
T	24	42.2601821	57.80851720	136.8%
Total	48	51.6680413	60.84415484	117.8%
C	24	244.9881891	167.34078836	68.3%
T	24	199.0005237	153.88557166	77.3%
Total	48	221.9943564	160.72334381	72.4%
C	72	103.8387776	144.98007184	139.6%
T	72	81.8973212	126.39433836	154.3%
Total	144	92.8680494	135.97507588	146.4%

Number of Levels	Covariance Structure	Number of Parameters	Subject Variables
1		1	
3		2	
2		1	
6		2	
1	Variance Components	1	ID
		1	
13		8	

r from those produced by prior versions. If you are using version 11 syntax, please consult the current

Denominator df	F	Sig.
42.000	39.097	.000
42.000	19.721	.000
42.000	.546	.464
42.000	.194	.825

Covariance Parameters[a]

Estimate	Std. Error	Wald Z	Sig.	95% Confidence Interval	
				Lower Bound	Upper Bound
284.077876	41.003110	6.928	.000	214.080471	376.962173
10493.784808	2310.635630	4.542	.000	6815.619610	16156.934497

Std. Error	df	95% Confidence Interval	
		Lower Bound	Upper Bound
25.725	42.000	-46.974	56.857
25.725	42.000	-.247	103.583
25.725	42.000	170.079	273.910

Std. Error	df	95% Confidence Interval	
		Lower Bound	Upper Bound
21.004	42.000	61.450	146.227
21.004	42.000	39.509	124.286

oup[a]

Mean	Std. Error	df	95% Confidence Interval	
			Lower Bound	Upper Bound
5.452	36.381	42.000	-67.967	78.872
4.431	36.381	42.000	-68.988	77.851
61.076	36.381	42.000	-12.343	134.495

42.260	36.381	42.000	-31.159	115.680
244.988	36.381	42.000	171.569	318.408
199.001	36.381	42.000	125.581	272.420

```
), ABSOLUTE) LCONVERGE(0, ABSOLUTE) PCONVERGE(0.000001, ABSOLUTE)
```

05-JUL-2016 15:16:21
J:\NYU Dental\Simone Duarte\Aline Castilho\ASP exp 1-4.sav
DataSet5
<none>
<none>
<none>
227
User-defined missing values are treated as missing.
Statistics are based on all cases with valid data for all variables in the model.

MIXED RASP BY Time Group<br>/CRITERIA=CIN(95) MXITER(100)<br>MXSTEP(10) SCORING(1)<br>SINGULAR(0.000000000001)<br>HCONVERGE(0, ABSOLUTE)<br>LCONVERGE(0, ABSOLUTE)<br>PCONVERGE(0.000001, ABSOLUTE)<br>/FIXED=Time Group Time*Group | SSTYPE(3) /METHOD=REML<br>/PRINT=DESCRIPTIVES SOLUTION<br>TESTCOV /RANDOM=INTERCEPT | SUBJECT(ID) COVTYPE(VC)<br>/EMMEANS=TABLES(Time)<br>/EMMEANS=TABLES(Group)<br>/EMMEANS=TABLES(Time*Group) .
00:00:00.02
00:00:00.05

atistics

	Count	Mean	Standard Deviation	Coefficient of Variation
T	3	33.00	0.000	0.0%
T	3	33.00	0.000	0.0%
C	3	33.00	0.000	0.0%
C	3	33.00	0.000	0.0%
T	3	33.00	0.000	0.0%
T	3	33.00	0.000	0.0%
C	3	33.00	0.000	0.0%
C	3	33.00	0.000	0.0%
T	3	33.00	0.000	0.0%

T	3	33.00	0.000	0.0%
C	3	33.00	0.000	0.0%
C	3	33.00	0.000	0.0%
T	3	33.00	0.000	0.0%
T	3	33.00	0.000	0.0%
C	3	33.00	0.000	0.0%
C	3	33.00	0.000	0.0%
T	3	33.00	0.000	0.0%
T	3	33.00	0.000	0.0%
C	3	33.00	0.000	0.0%
C	3	33.00	0.000	0.0%
T	3	129.67	4.726	3.6%
T	3	128.33	8.145	6.3%
C	3	118.33	1.528	1.3%
C	3	113.00	2.000	1.8%
T	3	33.00	0.000	0.0%
T	3	86.00	1.000	1.2%
C	3	74.00	3.000	4.1%
C	3	81.00	1.000	1.2%
T	3	91.33	1.528	1.7%
T	3	93.67	1.528	1.6%
C	3	109.00	1.000	.9%
C	3	105.67	1.528	1.4%
T	3	123.33	1.528	1.2%
T	3	139.67	1.528	1.1%
C	3	128.33	3.786	3.0%
C	3	143.00	1.000	.7%
T	3	69.67	4.726	6.8%
T	3	46.33	23.094	49.8%
C	3	70.33	1.528	2.2%
C	3	73.67	5.859	8.0%
T	3	107.33	7.767	7.2%
T	3	85.33	5.508	6.5%

C	3	99.33	3.055	3.1%
C	3	98.00	1.000	1.0%
T	3	90.00	11.269	12.5%
T	3	115.67	4.726	4.1%
C	3	129.67	3.055	2.4%
C	3	137.33	1.528	1.1%
C	24	53.88	21.602	40.1%
T	24	45.88	20.985	45.7%
Total	48	49.88	21.452	43.0%
C	24	68.00	35.918	52.8%
T	24	63.71	32.033	50.3%
Total	48	65.85	33.737	51.2%
C	24	104.46	43.146	41.3%
T	24	99.08	41.618	42.0%
Total	48	101.77	42.023	41.3%
C	72	75.44	40.406	53.6%
T	72	69.56	39.141	56.3%
Total	144	72.50	39.749	54.8%

Number of Levels	Covariance Structure	Number of Parameters	Subject Variables
1		1	
3		2	
2		1	
6		2	
1	Variance Components	1	ID
		1	
13		8	

r from those produced by prior versions. If you are using version 11 syntax, please consult the current

Denominator df	F	Sig.
42.000	205.345	.000
42.000	9.199	.000
42.000	.339	.564
42.000	.012	.988

Covariance Parameters[a]

Estimate	Std. Error	Wald Z	Sig.	95% Confidence Interval	
				Lower Bound	Upper Bound
20.590278	2.971951	6.928	.000	15.516789	27.322634
1221.797950	268.117723	4.557	.000	794.707441	1878.414813

Std. Error	df	95% Confidence Interval	
		Lower Bound	Upper Bound
8.763	42.000	32.190	67.560
8.763	42.000	48.170	83.539
8.763	42.000	84.086	119.455

Std. Error	df	95% Confidence Interval	
		Lower Bound	Upper Bound
7.155	42.000	61.005	89.884
7.155	42.000	55.116	83.995

oup[a]

Mean	Std. Error	df	95% Confidence Interval	
			Lower Bound	Upper Bound
53.875	12.393	42.000	28.865	78.885
45.875	12.393	42.000	20.865	70.885
68.000	12.393	42.000	42.990	93.010
63.708	12.393	42.000	38.699	88.718

	104.458	12.393	42.000	79.449	129.468
	99.083	12.393	42.000	74.074	124.093

05-JUL-2016 15:17:36
J:\NYU Dental\Simone Duarte\Aline Castilho\ASP exp 1-4.sav
DataSet5
<none>
<none>
<none>
227
GRAPH /LINE(MULTIPLE)=MEAN(RASP) BY Time BY Group /INTERVAL SE(1.0).
00:00:00.11
00:00:00.11

```
), ABSOLUTE) LCONVERGE(0, ABSOLUTE) PCONVERGE(0.000001, ABSOLUTE)
```

05-JUL-2016 15:35:59
J:\NYU Dental\Simone Duarte\Aline Castilho\ASP exp 1-4.sav
DataSet5
<none>
<none>
Time
227
User-defined missing values are treated as missing.
Statistics are based on all cases with valid data for all variables in the model.
MIXED RASP BY Group /CRITERIA=CIN(95) MXITER(100) MXSTEP(10) SCORING(1) SINGULAR(0.000000000001) HCONVERGE(0, ABSOLUTE) LCONVERGE(0, ABSOLUTE) PCONVERGE(0.000001, ABSOLUTE) /FIXED=Group | SSTYPE(3) /METHOD=REML /PRINT=DESCRIPTIVES SOLUTION TESTCOV   /RANDOM=INTERCEPT | SUBJECT(ID) COVTYPE(VC) /EMMEANS=TABLES(Group) .
00:00:00.00

	00:00:00.06

Count	Mean	Standard Deviation	Coefficient of Variation
3	33.00	0.000	0.0%
3	33.00	0.000	0.0%
3	33.00	0.000	0.0%
3	33.00	0.000	0.0%
3	33.00	0.000	0.0%
3	33.00	0.000	0.0%
3	33.00	0.000	0.0%
3	33.00	0.000	0.0%
3	33.00	0.000	0.0%
3	86.00	1.000	1.2%
3	74.00	3.000	4.1%
3	81.00	1.000	1.2%
3	69.67	4.726	6.8%
3	46.33	23.094	49.8%
3	70.33	1.528	2.2%
3	73.67	5.859	8.0%
24	53.88	21.602	40.1%
24	45.88	20.985	45.7%

48	49.88	21.452	43.0%

Number of Levels	Covariance Structure	Number of Parameters	Subject Variables
1		1	
2		1	
1	Variance Components	1	ID
		1	
4		4	

r from those produced by prior versions. If you are using version 11 syntax, please consult the current

Denominator df	F	Sig.
14	85.049	.000
14	.547	.472

Covariance Parameters[a,b]

Estimate	Std. Error	Wald Z	Sig.	95% Confidence Interval	
				Lower Bound	Upper Bound
37.708333	9.427083	4.000	.000	23.101328	61.551370
455.396825	176.902536	2.574	.010	212.683578	975.093004

Std. Error	df	95% Confidence Interval	
		Lower Bound	Upper Bound

7.648	14	37.471	70.279
7.648	14	29.471	62.279

Count	Mean	Standard Deviation	Coefficient of Variation
3	33.00	0.000	0.0%
3	33.00	0.000	0.0%
3	33.00	0.000	0.0%
3	33.00	0.000	0.0%
3	33.00	0.000	0.0%
3	33.00	0.000	0.0%
3	33.00	0.000	0.0%
3	33.00	0.000	0.0%
3	91.33	1.528	1.7%
3	93.67	1.528	1.6%
3	109.00	1.000	.9%
3	105.67	1.528	1.4%
3	107.33	7.767	7.2%
3	85.33	5.508	6.5%
3	99.33	3.055	3.1%
3	98.00	1.000	1.0%
24	68.00	35.918	52.8%
24	63.71	32.033	50.3%
48	65.85	33.737	51.2%

Number of Levels	Covariance Structure	Number of Parameters	Subject Variables
1		1	
2		1	
1	Variance Components	1	ID
		1	
4		4	

ʳ from those produced by prior versions. If you are using version 11 syntax, please consult the current

Denominator df	F	Sig.
14.000	54.930	.000
14.000	.058	.813

Covariance Parameters[a,b]

Estimate	Std. Error	Wald Z	Sig.	95% Confidence Interval	
				Lower Bound	Upper Bound
6.812500	1.703125	4.000	.000	4.173555	11.120054
1260.942460	477.450084	2.641	.008	600.332293	2648.493021

Std. Error	df	95% Confidence Interval	
		Lower Bound	Upper Bound
12.566	14.000	41.049	94.951
12.566	14.000	36.757	90.659

Count	Mean	Standard Deviation	Coefficient of Variation
3	33.00	0.000	0.0%
3	33.00	0.000	0.0%
3	33.00	0.000	0.0%
3	33.00	0.000	0.0%
3	129.67	4.726	3.6%
3	128.33	8.145	6.3%
3	118.33	1.528	1.3%
3	113.00	2.000	1.8%
3	123.33	1.528	1.2%
3	139.67	1.528	1.1%
3	128.33	3.786	3.0%
3	143.00	1.000	.7%
3	90.00	11.269	12.5%
3	115.67	4.726	4.1%
3	129.67	3.055	2.4%
3	137.33	1.528	1.1%
24	104.46	43.146	41.3%
24	99.08	41.618	42.0%
48	101.77	42.023	41.3%

Number of Levels	Covariance Structure	Number of Parameters	Subject Variables
1		1	
2		1	
1	Variance Components	1	ID
		1	
4		4	

r from those produced by prior versions. If you are using version 11 syntax, please consult the current

Denominator df	F	Sig.
14	84.774	.000
14	.059	.811

Covariance Parameters[a,b]

Estimate	Std. Error	Wald Z	Sig.	95% Confidence Interval	
				Lower Bound	Upper Bound
17.250000	4.312500	4.000	.000	10.567900	28.157201
1949.054563	738.848075	2.638	.008	927.150657	4097.299249

Std. Error	df	95% Confidence Interval	
		Lower Bound	Upper Bound
15.632	14	70.932	137.985
15.632	14	65.557	132.610
