# Supplementary material for: Trans,trans-farnesol, an antimicrobial natural compound, improves glass ionomer cement properties
Source: PLoS One. 2019 Aug 20;14(8):e0220718. doi: 10.1371/journal.pone.0220718 (PMC6701760; doi:10.1371/journal.pone.0220718)
Supplement: S11 Text — (PDF) [file pone.0220718.s015.pdf]

```
DATASET ACTIVATE DataSet9.

SAVE OUTFILE='E:\NYU Dental\Simone Duarte\Aline Castilho\CFU exp 1-4.sav'
  /COMPRESSED.
GRAPH
  /LINE(MULTIPLE)=MEAN(log10.cfu) BY Day BY Group
  /INTERVAL SE(1).
```

Graph

Notes

Output Created		30-JUN-2016 15:39:45
Comments		
Input	Data	E:\NYU Dental\Simone Duarte\Aline Castilho\CFU exp 1-4.sav
	Active Dataset	DataSet9
	Filter	<none>
	Weight	<none>
	Split File	<none>
	N of Rows in Working Data File	100
Syntax		GRAPH<br>/LINE(MULTIPLE)=MEAN(log10.cfu) BY Day BY Group<br>/INTERVAL SE(1).
Resources	Processor Time	00:00:00.14
	Elapsed Time	00:00:00.31

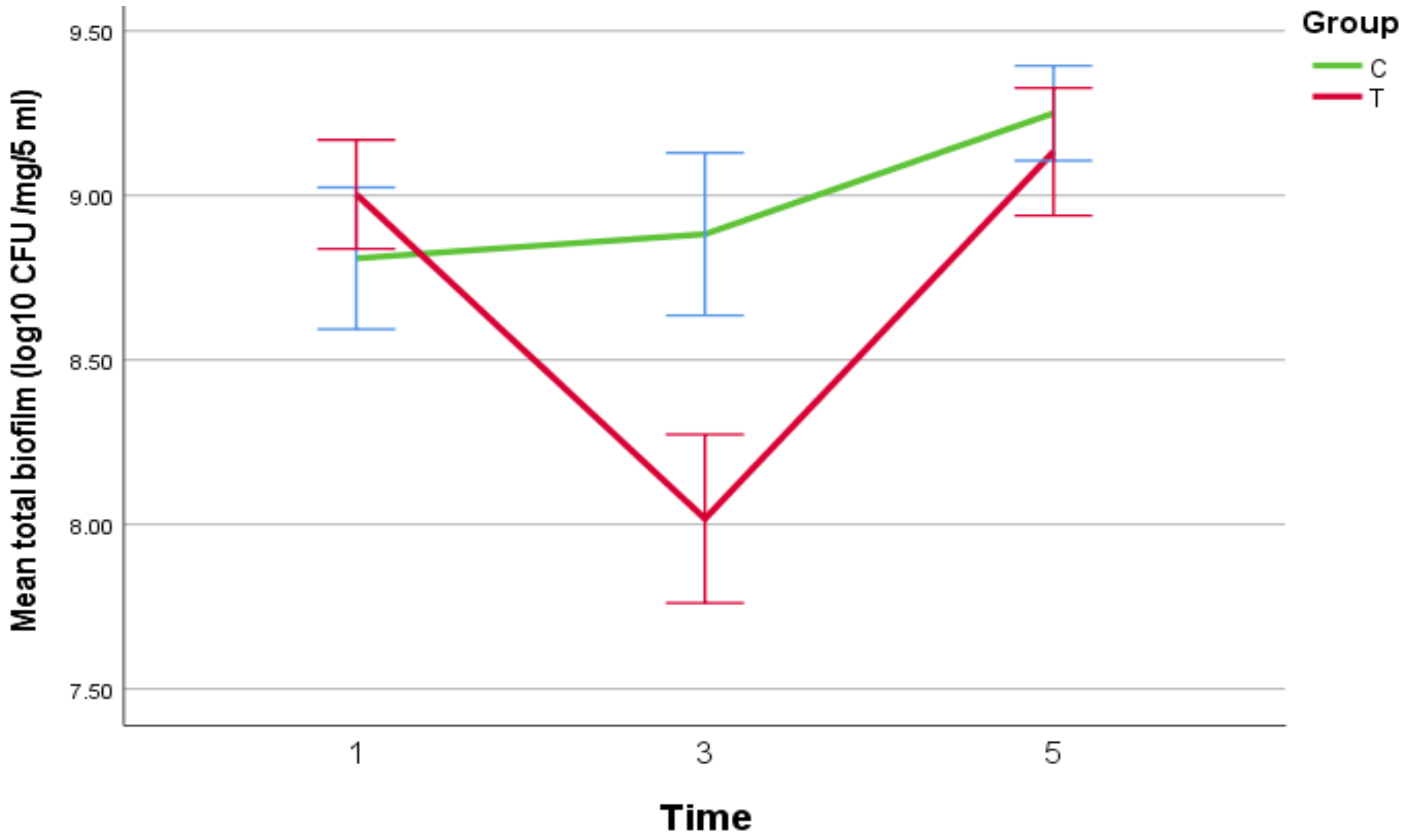

Error bars: +/- 1 SE

```
MIXED log10.cfu BY Group Day
  /CRITERIA=CIN(95) MXITER(100) MXSTEP(10) SCORING(1) SINGULAR(0.000000000001) HCONVERGE(0, ABSOLUTE) LCONVI
  /FIXED=Group Day Group*Day | SSTYPE(3)
  /METHOD=REML
  /PRINT=DESCRIPTIVES  SOLUTION TESTCOV
  /EMMEANS=TABLES(Group)
  /EMMEANS=TABLES(Day)
  /EMMEANS=TABLES(Group*Day) .
```

Mixed Model Analysis

Notes

Output Created		30-JUN-2016 15:40:51
Comments		
Input	Data	E:\NYU Dental\Simone Duarte\Aline Castilho\CFU exp 1-4.sav
	Active Dataset	DataSet9
	Filter	<none>
	Weight	<none>
	Split File	<none>
	N of Rows in Working Data File	100
Missing Value Handling	Definition of Missing	User-defined missing values are treated as missing.
	Cases Used	Statistics are based on all cases with valid data for all variables in the model.

Syntax		MIXED log10.cfu BY Group Day<br>/CRITERIA=CIN(95) MXITER(100) MXSTEP(10) SCORING(1) SINGULAR(0.000000000001) HCONVERGE(0, ABSOLUTE) LCONVERGE(0, ABSOLUTE) PCONVERGE(0.000001, ABSOLUTE)<br>/FIXED=Group Day Group*Day | SSTYPE(3)<br>/METHOD=REML<br>/PRINT=DESCRIPTIVES SOLUTION TESTCOV<br>/EMMEANS=TABLES(Group)<br>/EMMEANS=TABLES(Day)<br>/EMMEANS=TABLES(Group*Day) .
Resources	Processor Time	00:00:00.02
	Elapsed Time	00:00:00.01

Descriptive Statistics

total biofilm (log10 CFU /mg/5 ml)

Group		Count	Mean	Standard Deviation	Coefficient of Variation
C	1	8	8.8087	.60930	6.9%
	3	8	8.8822	.69883	7.9%
	5	8	9.2497	.40784	4.4%
	Total	24	8.9802	.59252	6.6%
T	1	8	9.0032	.46827	5.2%
	3	8	8.0173	.72444	9.0%
	5	8	9.1326	.54851	6.0%
	Total	24	8.7177	.75952	8.7%
Total	1	16	8.9060	.53447	6.0%

	3		16	8.4498	.81993	9.7%
	5		16	9.1912	.47083	5.1%
	Total		48	8.8490	.68680	7.8%

Model Dimension[a]

		Number of Levels	Number of Parameters
Fixed Effects	Intercept	1	1
	Group	2	1
	Day	3	2
	Group * Day	6	2
Residual			1
Total		12	7

a. Dependent Variable: total biofilm (log10 CFU /mg/5 ml).

Information Criteria[a]

-2 Restricted Log Likelihood	86.989
Akaike's Information Criterion (AIC)	88.989
Hurvich and Tsai's Criterion (AICC)	89.089
Bozdogan's Criterion (CAIC)	91.726
Schwarz's Bayesian Criterion (BIC)	90.726

The information criteria are displayed in smaller-is-better form.

a. Dependent Variable: total biofilm (log10 CFU /mg/5 ml).

Fixed Effects

Type III Tests of Fixed Effects[a]

Source	Numerator df	Denominator df	F	Sig.
Intercept	1	42	10889.834	.000
Group	1	42	2.396	.129
Day	2	42	6.483	.004
Group * Day	2	42	3.435	.042

a. Dependent Variable: total biofilm (log10 CFU /mg/5 ml).

Covariance Parameters

Estimates of Covariance Parameters[a]

					95% Confide
Parameter	Estimate	Std. Error	Wald Z	Sig.	Lower Bound
Residual	.345149	.075318	4.583	.000	.225039

a. Dependent Variable: total biofilm (log10 CFU /mg/5 ml).

Estimated Marginal Means

1. Group[a]

				95% Confidence Interval	

Group	Mean	Std. Error	df	Lower Bound	Upper Bound
C	8.980	.120	42	8.738	9.222
T	8.718	.120	42	8.476	8.960

a. Dependent Variable: total biofilm (log10 CFU /mg/5 ml).

2. Day[a]

Day	Mean	Std. Error	df	95% Confidence Interval	
				Lower Bound	Upper Bound
1	8.906	.147	42	8.610	9.202
3	8.450	.147	42	8.153	8.746
5	9.191	.147	42	8.895	9.488

a. Dependent Variable: total biofilm (log10 CFU /mg/5 ml).

3. Group * Day[a]

Group		Mean	Std. Error	df	95% Confide
					Lower Bound
C	1	8.809	.208	42	8.390
	3	8.882	.208	42	8.463
	5	9.250	.208	42	8.831
T	1	9.003	.208	42	8.584
	3	8.017	.208	42	7.598
	5	9.133	.208	42	8.713

a. Dependent Variable: total biofilm (log10 CFU /mg/5 ml).

ERGE(0, ABSOLUTE) PCONVERGE(0.000001, ABSOLUTE)

ence Interval
Upper Bound
.529363

ence Interval
Upper Bound
9.228
9.301
9.669
9.422
8.437
9.552
