# Supplementary material for: Trans,trans-farnesol, an antimicrobial natural compound, improves glass ionomer cement properties
Source: PLoS One. 2019 Aug 20;14(8):e0220718. doi: 10.1371/journal.pone.0220718 (PMC6701760; doi:10.1371/journal.pone.0220718)
Supplement: S12 Text — (PDF) [file pone.0220718.s016.pdf]

```
DATASET ACTIVATE DataSet8.

SAVE OUTFILE='J:\NYU Dental\Simone Duarte\Aline Castilho\Volume exp 1-4.sav'
  /COMPRESSED.
GRAPH
  /LINE(MULTIPLE)=MEAN(DW) BY Day BY Group
  /INTERVAL SE(1).
```

Graph

Notes

Output Created		05-JUL-2016 15:59:22
Comments		
Input	Data	J:\NYU Dental\Simone Duarte\Aline Castilho\Volume exp 1-4.sav
	Active Dataset	DataSet8
	Filter	<none>
	Weight	<none>
	Split File	<none>
	N of Rows in Working Data File	116
Syntax		GRAPH /LINE(MULTIPLE)=MEAN(DW) BY Day BY Group /INTERVAL SE(1).
Resources	Processor Time	00:00:00.09
	Elapsed Time	00:00:00.09

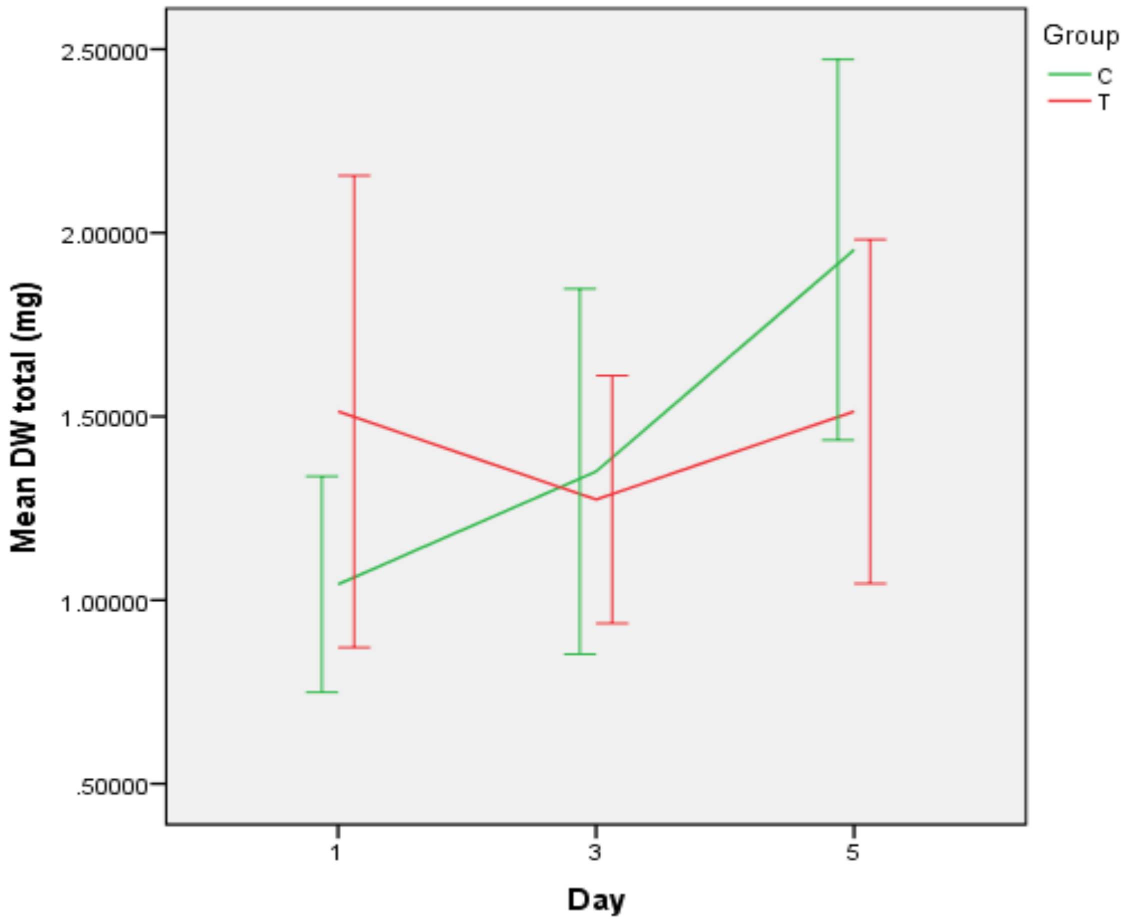

Error bars: +/- 1 SE

```
MIXED DW BY Day Group
  /CRITERIA=CIN(95) MXITER(100) MXSTEP(10) SCORING(1) SINGULAR(0.000000000001) HCONVERGE(0, ABSOLUTE) L[illegible]
  /FIXED=Day Group Day*Group | SSTYPE(3)
  /METHOD=REML
  /PRINT=DESCRIPTIVES  SOLUTION TESTCOV
  /EMMEANS=TABLES(Day)
  /EMMEANS=TABLES(Group)
  /EMMEANS=TABLES(Day*Group) .
```

Mixed Model Analysis

Notes

Output Created		05-JUL-2016 16:00:31
Comments		
Input	Data	J:\NYU Dental\Simone Duarte\Aline Castilho\Volume exp 1-4.sav
	Active Dataset	DataSet8
	Filter	<none>
	Weight	<none>
	Split File	<none>
	N of Rows in Working Data File	116
Missing Value Handling	Definition of Missing	User-defined missing values are treated as missing.
	Cases Used	Statistics are based on all cases with valid data for all variables in the model.
Syntax		MIXED DW BY Day Group /CRITERIA=CIN(95) MXITER(100) MXSTEP(10) SCORING(1) SINGULAR(0.000000000001) HCONVERGE(0, ABSOLUTE) LCONVERGE(0, ABSOLUTE) PCONVERGE(0.000001, ABSOLUTE) /FIXED=Day Group Day*Group	SSTYPE(3) /METHOD=REML /PRINT=DESCRIPTIVES SOLUTION TESTCOV /EMMEANS=TABLES(Day) /EMMEANS=TABLES(Group) /EMMEANS=TABLES(Day*Group) .
Resources	Processor Time	00:00:00.00

Elapsed Time	00:00:00.02

Model Dimension[a]

		Number of Levels	Number of Parameters
Fixed Effects	Intercept	1	1
	Day	3	2
	Group	2	1
	Day * Group	6	2
Residual			1
Total		12	7

a. Dependent Variable: DW total (mg).

Information Criteria[a]

-2 Restricted Log Likelihood	168.242
Akaike's Information Criterion (AIC)	170.242
Hurvich and Tsai's Criterion (AICC)	170.338
Bozdogan's Criterion (CAIC)	173.027
Schwarz's Bayesian Criterion (BIC)	172.027

The information criteria are displayed in smaller-is-better form.

a. Dependent Variable: DW total (mg).

Fixed Effects

Type III Tests of Fixed Effects[a]

Source	Numerator df	Denominator df	F
Intercept	1	44	51.285
Day	2	44	.535
Group	1	44	.001
Day * Group	2	44	.425

a. Dependent Variable: DW total (mg).

Covariance Parameters

Estimates of Covariance Parameters[a]

Parameter	Estimate	Std. Error	Wald Z
Residual	2.008235	.428157	4.690

a. Dependent Variable: DW total (mg).

Estimated Marginal Means

1. Day[a]

Day	Mean	Std. Error	df
1	1.278	.357	44
3	1.312	.344	44
5	1.734	.344	44

a. Dependent Variable: DW total (mg).

2. Group[a]

Group	Mean	Std. Error	df
C	1.449	.291	44

T	1.434	.278	44

a. Dependent Variable: DW total (mg).

3. Day * Group[a]

Day		Mean	Std. Error
1	C	1.043	.536
	T	1.514	.472
3	C	1.350	.472
	T	1.274	.501
5	C	1.954	.501
	T	1.514	.472

a. Dependent Variable: DW total (mg).

```
EXAMINE VARIABLES=DW BY Day BY Group
  /PLOT=BOXPLOT
  /STATISTICS=NONE
  /NOTOTAL.
```

Explore

Notes

Output Created		05-JUL-2016 16:01:47
Comments		
Input	Data	J:\NYU Dental\Simone Duarte\Aline Castilho\Volume exp 1-4.sav
	Active Dataset	DataSet8
	Filter	<none>
	Weight	<none>
	Split File	<none>
	N of Rows in Working Data File	116

Missing Value Handling	Definition of Missing	User-defined missing values for dependent variables are treated as missing.
	Cases Used	Statistics are based on cases with no missing values for any dependent variable or factor used.
Syntax		EXAMINE VARIABLES=DW BY Day BY Group /PLOT=BOXPLOT /STATISTICS=NONE /NOTOTAL.
Resources	Processor Time	00:00:00.09
	Elapsed Time	00:00:00.11

Day*Group

Case Processing Summary

			Valid
Day			N
DW total (mg)	1	C	7
		T	9
	3	C	9
		T	8
	5	C	8
		T	9

DW total (mg)

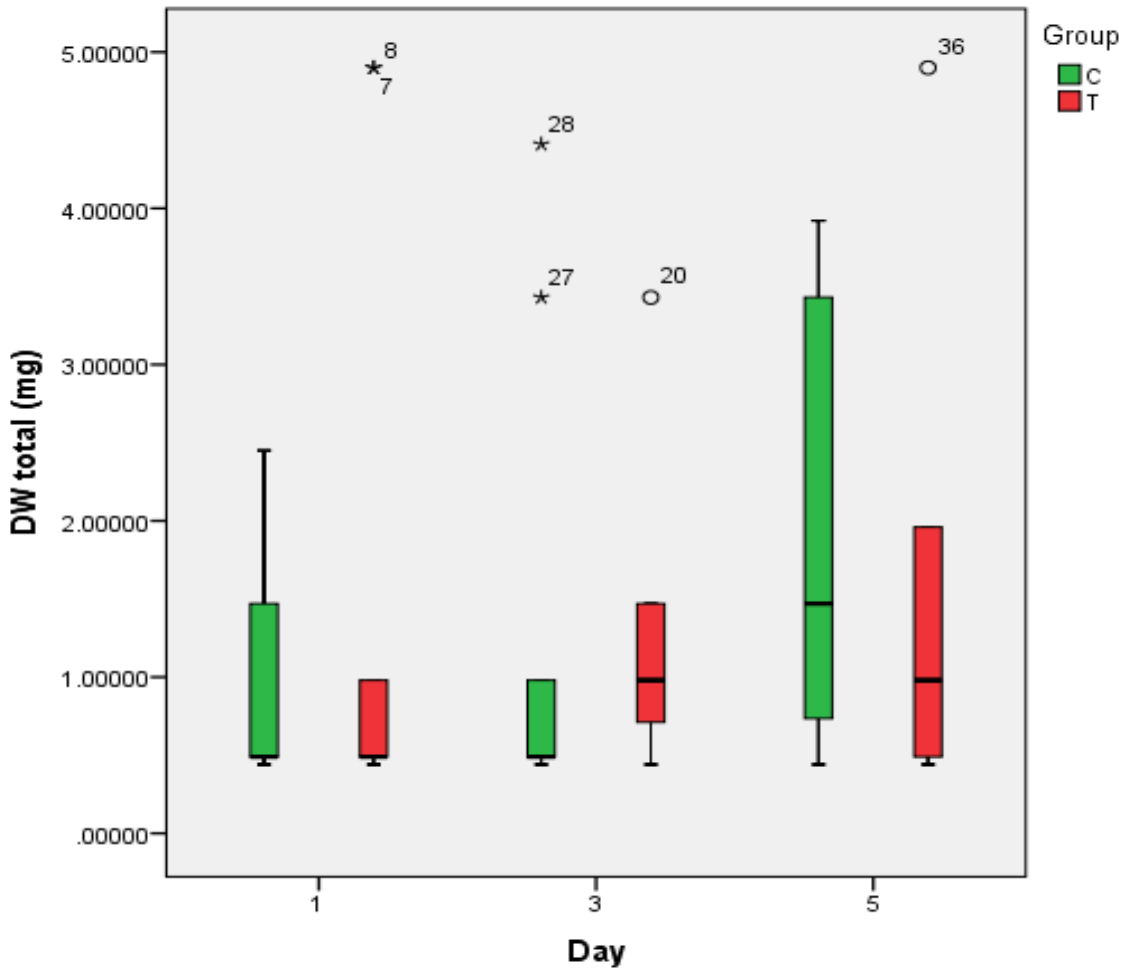


```
RANK VARIABLES=DW (A)
  /RANK
  /PRINT=YES
  /TIES=MEAN.
```

RANK

Notes

Output Created		05-JUL-2016 16:02:08
Comments		
Input	Data	J:\NYU Dental\Simone Duarte\Aline Castilho\Volume exp 1-4.sav
	Active Dataset	DataSet8
	Filter	<none>
	Weight	<none>
	Split File	<none>
	N of Rows in Working Data File	116
Missing Value Handling	Definition of Missing	User-defined missing values are treated as missing.
	Cases Used	All non-missing data are used.
Syntax		RANK VARIABLES=DW (A) /RANK /PRINT=YES /TIES=MEAN.
Resources	Processor Time	00:00:00.00
	Elapsed Time	00:00:00.00
Variables Created or Modified	RDW	Rank of DW

Created Variables[a]

Source Variable		New Variable	Label
DW[b]	Rank	RDW	Rank of DW

a. Mean rank of tied values is used for ties.

b. Ranks are in ascending order.

```
MIXED RDW BY Day Group
  /CRITERIA=CIN(95) MXITER(100) MXSTEP(10) SCORING(1) SINGULAR(0.000000000001) HCONVERGE(0, ABSOLUTE) L(
  /FIXED=Day Group Day*Group | SSTYPE(3)
  /METHOD=REML
  /PRINT=DESCRIPTIVES  SOLUTION TESTCOV
```

```
/EMMEANS=TABLES(Day)
/EMMEANS=TABLES(Group)
/EMMEANS=TABLES(Day*Group) .
```

Mixed Model Analysis

Notes

Output Created		05-JUL-2016 16:02:21
Comments		
Input	Data	J:\NYU Dental\Simone Duarte\Aline Castilho\Volume exp 1-4.sav
	Active Dataset	DataSet8
	Filter	<none>
	Weight	<none>
	Split File	<none>
	N of Rows in Working Data File	116
Missing Value Handling	Definition of Missing	User-defined missing values are treated as missing.
	Cases Used	Statistics are based on all cases with valid data for all variables in the model.

Syntax		MIXED RDW BY Day Group /CRITERIA=CIN(95) MXITER(100) MXSTEP(10) SCORING(1) SINGULAR(0.000000000001) HCONVERGE(0, ABSOLUTE) LCONVERGE(0, ABSOLUTE) PCONVERGE(0.000001, ABSOLUTE) /FIXED=Day Group Day*Group | SSTYPE(3) /METHOD=REML /PRINT=DESCRIPTIVES SOLUTION TESTCOV /EMMEANS=TABLES(Day) /EMMEANS=TABLES(Group) /EMMEANS=TABLES(Day*Group) .
Resources	Processor Time	00:00:00.00
	Elapsed Time	00:00:00.02

Descriptive Statistics

Rank of DW

Day		Count	Mean
1	C	7	23.71429
	T	9	22.50000
	Total	16	23.03125
3	C	9	22.88889
	T	8	25.87500
	Total	17	24.29412
5	C	8	31.12500
	T	9	27.16667
	Total	17	29.02941
Total	C	24	25.87500
	T	26	25.15385
	Total	50	25.50000

Model Dimension[a]

		Number of Levels	Number of Parameters
Fixed Effects	Intercept	1	1
	Day	3	2
	Group	2	1
	Day * Group	6	2
Residual			1
Total		12	7

a. Dependent Variable: Rank of DW.

Information Criteria[a]

-2 Restricted Log Likelihood	374.726
Akaike's Information Criterion (AIC)	376.726
Hurvich and Tsai's Criterion (AICC)	376.821
Bozdogan's Criterion (CAIC)	379.510
Schwarz's Bayesian Criterion (BIC)	378.510

The information criteria are displayed in smaller-is-better form.

a. Dependent Variable: Rank of DW.

Fixed Effects

Type III Tests of Fixed Effects[a]

Source	Numerator df	Denominator df	F
Intercept	1	44	147.566

Day	2	44	.766
Group	1	44	.030
Day * Group	2	44	.236

a. Dependent Variable: Rank of DW.

Covariance Parameters

Estimates of Covariance Parameters[a]

Parameter	Estimate	Std. Error	Wald Z
Residual	219.217442	46.737316	4.690

a. Dependent Variable: Rank of DW.

Estimated Marginal Means

1. Day[a]

Day	Mean	Std. Error	df
1	23.107	3.731	44
3	24.382	3.597	44
5	29.146	3.597	44

a. Dependent Variable: Rank of DW.

2. Group[a]

Group	Mean	Std. Error	df
C	25.909	3.038	44
T	25.181	2.908	44

a. Dependent Variable: Rank of DW.

3. Day * Group[a]

Day		Mean	Std. Error
1	C	23.714	5.596
	T	22.500	4.935
3	C	22.889	4.935
	T	25.875	5.235
5	C	31.125	5.235
	T	27.167	4.935

a. Dependent Variable: Rank of DW.

```
CONVERGE(0, ABSOLUTE) PCONVERGE(0.000001, ABSOLUTE)
```

Sig.
.000
.589
.970
.656

Sig.	95% Confidence Interval	
	Lower Bound	Upper Bound
.000	1.322323	3.049940

95% Confidence Interval	
Lower Bound	Upper Bound
.559	1.998
.618	2.006
1.040	2.428

95% Confidence Interval	
Lower Bound	Upper Bound
.863	2.035

.873	1.995

df	95% Confidence Interval	
	Lower Bound	Upper Bound
44	-.036	2.122
44	.562	2.466
44	.398	2.302
44	.264	2.284
44	.944	2.964
44	.562	2.466

Cases					
	Missing		Total		
Percent	N	Percent	N	Percent
100.0%	0	0.0%	7	100.0%
100.0%	0	0.0%	9	100.0%
100.0%	0	0.0%	9	100.0%
100.0%	0	0.0%	8	100.0%
100.0%	0	0.0%	8	100.0%
100.0%	0	0.0%	9	100.0%

```
CONVERGE(0, ABSOLUTE) PCONVERGE(0.000001, ABSOLUTE)
```

Standard Deviation	Coefficient of Variation
13.174651	55.6%
16.399314	72.9%
14.603046	63.4%
14.899198	65.1%
13.708053	53.0%
13.984432	57.6%
14.591460	46.9%
15.292972	56.3%
14.636716	50.4%
14.205212	54.9%
14.753826	58.7%
14.349500	56.3%

Sig.
.000

.471
.863
.791

Sig.	95% Confidence Interval	
	Lower Bound	Upper Bound
.000	144.343864	332.929198

95% Confidence Interval	
Lower Bound	Upper Bound
15.588	30.626
17.132	31.632
21.896	36.396

95% Confidence Interval	
Lower Bound	Upper Bound
19.786	32.033
19.320	31.042

df	95% Confidence Interval	
	Lower Bound	Upper Bound
44	12.436	34.993
44	12.553	32.447
44	12.942	32.835
44	15.325	36.425
44	20.575	41.675
44	17.220	37.113
