# Supplementary material for: Trans,trans-farnesol, an antimicrobial natural compound, improves glass ionomer cement properties
Source: PLoS One. 2019 Aug 20;14(8):e0220718. doi: 10.1371/journal.pone.0220718 (PMC6701760; doi:10.1371/journal.pone.0220718)
Supplement: S13 Text — (PDF) [file pone.0220718.s017.pdf]

```
DATASET ACTIVATE DataSet1.
DATASET CLOSE DataSet2.
GET DATA /TYPE=XLSX
  /FILE='J:\NYU Dental\Simone Duarte\Aline Castilho\Table biofilm Castilho mj.xlsx'
  /SHEET=name 'exp 1-4 WSP'
  /CELLRANGE=full
  /READNAMES=on
  /ASSUMEDSTRWIDTH=32767.
EXECUTE.
DATASET NAME DataSet3 WINDOW=FRONT.

SAVE OUTFILE='J:\NYU Dental\Simone Duarte\Aline Castilho\WSp exp 1-4.sav'
  /COMPRESSED.
GRAPH
  /LINE(MULTIPLE)=MEAN(WSP) BY Time BY Group
  /INTERVAL SE(1).
```

Graph

Notes

Output Created		05-JUL-2016 15:02:16
Comments		
Input	Data	J:\NYU Dental\Simone Duarte\Aline Castilho\WSp exp 1-4.sav
	Active Dataset	DataSet3
	Filter	<none>
	Weight	<none>
	Split File	<none>
	N of Rows in Working Data File	304
Syntax		GRAPH /LINE(MULTIPLE)=MEAN(WSP) BY Time BY Group /INTERVAL SE(1).

Resources	Processor Time	00:00:00.27
	Elapsed Time	00:00:00.66

[DataSet3] J:\NYU Dental\Simone Duarte\Aline Castilho\WSp exp 1-4.sav

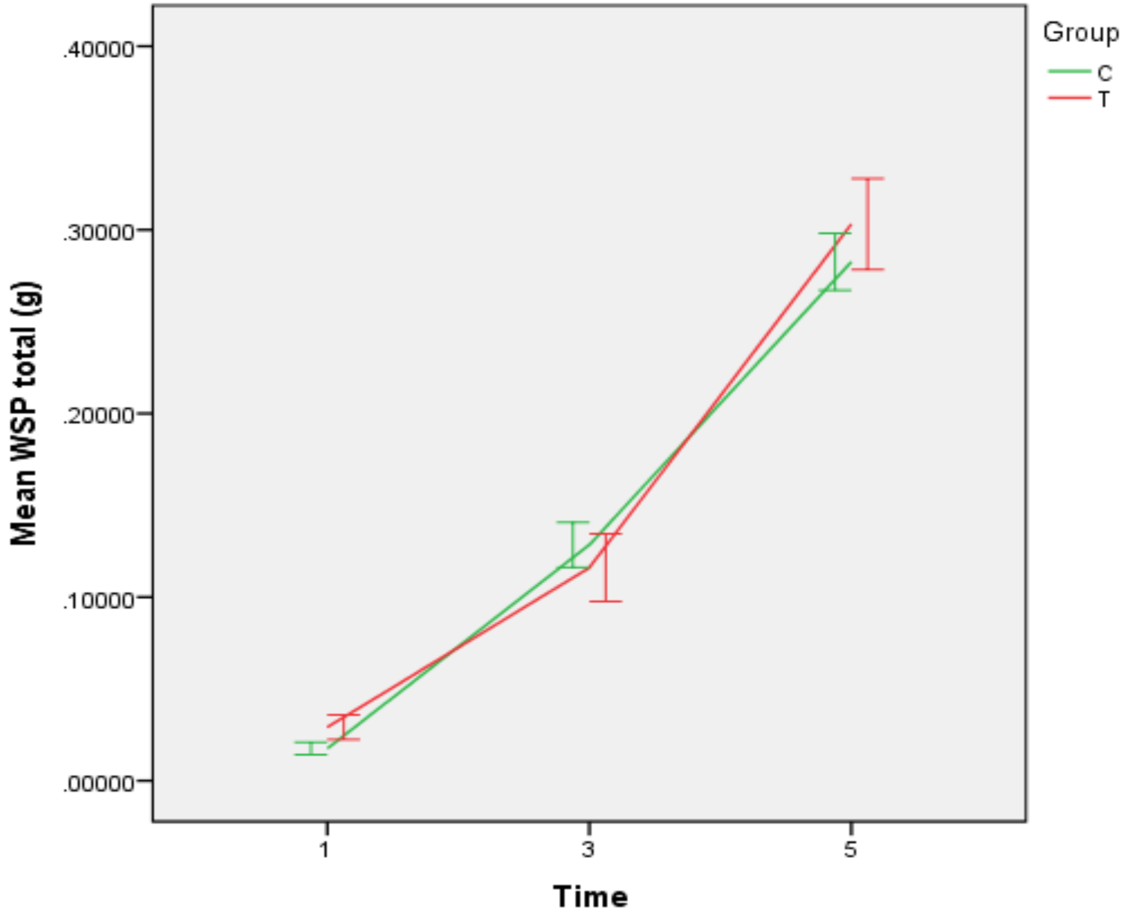

Error bars: +/- 1 SE

```
MIXED WSP BY Time Group
  /CRITERIA=CIN(95) MXITER(100) MXSTEP(10) SCORING(1) SINGULAR(0.000000000001) HCONVERGE(0, ABSOLUTE) [illegible]
  /FIXED=Time Group Time*Group | SSTYPE(3)
```

```
/METHOD=REML
/PRINT=DESCRIPTIVES  SOLUTION TESTCOV
/RANDOM=INTERCEPT | SUBJECT(ID) COVTYPE(VC)
/EMMEANS=TABLES(Time)
/EMMEANS=TABLES(Group)
/EMMEANS=TABLES(Time*Group) .
```

Mixed Model Analysis

Notes

Output Created		05-JUL-2016 15:03:01
Comments		
Input	Data	J:\NYU Dental\Simone Duarte\Aline Castilho\WSp exp 1-4.sav
	Active Dataset	DataSet3
	Filter	<none>
	Weight	<none>
	Split File	<none>
	N of Rows in Working Data File	304
Missing Value Handling	Definition of Missing	User-defined missing values are treated as missing.
	Cases Used	Statistics are based on all cases with valid data for all variables in the model.

Syntax		MIXED WSP BY Time Group /CRITERIA=CIN(95) MXITER(100) MXSTEP(10) SCORING(1) SINGULAR(0.000000000001) HCONVERGE(0, ABSOLUTE) LCONVERGE(0, ABSOLUTE) PCONVERGE(0.000001, ABSOLUTE) /FIXED=Time Group Time*Group | SSTYPE(3) /METHOD=REML /PRINT=DESCRIPTIVES SOLUTION TESTCOV /RANDOM=INTERCEPT | SUBJECT(ID) COVTYPE(VC) /EMMEANS=TABLES(Time) /EMMEANS=TABLES(Group) /EMMEANS=TABLES(Time*Group) .
Resources	Processor Time	00:00:00.03
	Elapsed Time	00:00:00.06

Descriptive Statistics

WSP total (g)

ID			Count	Mean
1	1	T	3	.0863782
2	1	T	3	.0802564
3	1	C	3	.0337607
4	1	C	3	.0355235
5	3	T	3	.2089637
6	3	T	3	.2706410
7	3	C	3	.1687179
8	3	C	3	.2258868
9	5	T	3	.3012393

10	5	T	3	.3229380
11	5	C	3	.3299359
12	5	C	3	.3231410
13	1	T	3	.0066346
14	1	T	3	.0157265
15	1	C	3	.0159829
16	1	C	3	.0095940
17	3	T	3	.0100855
18	3	T	3	.0243162
19	3	C	3	.0498077
20	3	C	3	.0534295
21	5	T	3	.4207372
22	5	T	3	.4474252
23	5	C	3	.2159829
24	5	C	2	.1998878
25	1	T	3	.0130556
26	1	T	3	.0219231
27	1	C	3	.0015919
28	1	C	3	.0197863
29	3	T	3	.1055128
30	3	T	3	.1560470
31	3	C	3	.1726603
32	3	C	3	.1095085
33	5	T	3	.3001175
34	5	T	3	.3236325
35	5	C	3	.4181197
36	5	C	3	.2671688
37	1	T	3	.0040278
38	1	T	3	.0047970
39	1	C	3	.0126816
40	1	C	3	.0116453
41	3	T	3	.1146902
42	3	T	3	.0380235

43	3	C	3	.1419872
44	3	C	3	.1058440
45	5	T	3	.0512393
46	5	T	3	.2581838
47	5	C	3	.2807051
48	5	C	3	.1987393
Total	1	C	24	.0175708
		T	24	.0290999
		Total	48	.0233353
	3	C	24	.1284802
		T	24	.1160350
		Total	48	.1222576
	5	C	23	.2826589
		T	24	.3031891
		Total	47	.2931424
	Total	C	71	.1409349
		T	72	.1494413
		Total	143	.1452179

Totals that are aggregated over either a single category of a variable or a split file variable are omitted.

Model Dimension[a]

		Number of Levels	Covariance Structure	Number of Parameters
Fixed Effects	Intercept	1		1
	Time	3		2
	Group	2		1
	Time * Group	6		2
Random Effects	Intercept[b]	1	Variance Components	1
Residual				1
Total		13		8

a. Dependent Variable: WSP total (g).

b. As of version 11.5, the syntax rules for the RANDOM subcommand have changed. Your command syntax may yield results that differ from those produc
versions. If you are using version 11 syntax, please consult the current syntax reference guide for more information.

Information Criteria[a]

-2 Restricted Log Likelihood	-465.216
Akaike's Information Criterion (AIC)	-461.216
Hurvich and Tsai's Criterion (AICC)	-461.127
Bozdogan's Criterion (CAIC)	-453.376
Schwarz's Bayesian Criterion (BIC)	-455.376

The information criteria are displayed in smaller-is-better form.

a. Dependent Variable: WSP total (g).

Fixed Effects

Type III Tests of Fixed Effects[a]

Source	Numerator df	Denominator df	F	Sig.
Intercept	1	41.992	180.701	.000
Time	2	41.992	52.130	.000
Group	1	41.992	.124	.727
Time * Group	2	41.992	.241	.787

a. Dependent Variable: WSP total (g).

Covariance Parameters

Estimates of Covariance Parameters[a]

Parameter		Estimate	Std. Error	Wald Z
Residual		.000622	9.030335E-05	6.892
Intercept [subject = ID]	Variance	.005424	.001230	4.410

a. Dependent Variable: WSP total (g).

Estimated Marginal Means

1. Time[a]

Time	Mean	Std. Error	df	95% Confide
				Lower Bound
1	.023	.019	41.961	-.015
3	.122	.019	41.961	.084
5	.291	.019	42.054	.253

a. Dependent Variable: WSP total (g).

2. Group[a]

Group	Mean	Std. Error	df	95% Confide
				Lower Bound
C	.142	.015	42.023	.111
T	.149	.015	41.961	.119

a. Dependent Variable: WSP total (g).

3. Time * Group[a]

Time		Mean	Std. Error	df
1	C	.018	.027	41.961
	T	.029	.027	41.961
3	C	.128	.027	41.961
	T	.116	.027	41.961

5	C		.279	.027	42.148
	T		.303	.027	41.961

a. Dependent Variable: WSP total (g).

LCONVERGE(0, ABSOLUTE) PCONVERGE(0.000001, ABSOLUTE)

Standard Deviation	Coefficient of Variation
.00761506	8.8%
.00571889	7.1%
.01343719	39.8%
.03580622	100.8%
.00725927	3.5%
.05568479	20.6%
.04796972	28.4%
.01226439	5.4%
.01243723	4.1%

.02340403	7.2%
.01301314	3.9%
.02354709	7.3%
.00167497	25.2%
.00558294	35.5%
.00164724	10.3%
.00590312	61.5%
.00206832	20.5%
.00132707	5.5%
.00136322	2.7%
.00547664	10.3%
.03554520	8.4%
.02219352	5.0%
.01162120	5.4%
.01498069	7.5%
.00826428	63.3%
.00019496	.9%
.00929898	584.2%
.00081736	4.1%
.00281705	2.7%
.00428533	2.7%
.01528275	8.9%
.00273946	2.5%
.03098851	10.3%
.11269049	34.8%
.02240278	5.4%
.00941202	3.5%
.00040837	10.1%
.00150891	31.5%
.00399708	31.5%
.00382168	32.8%
.00752587	6.6%
.00406045	10.7%

.00837588	5.9%
.00465189	4.4%
.02103854	41.1%
.04334168	16.8%
.03723130	13.3%
.01689018	8.5%
.01634584	93.0%
.03278348	112.7%
.02627989	112.6%
.06023130	46.9%
.09003500	77.6%
.07603795	62.2%
.07447834	26.3%
.12153348	40.1%
.10072603	34.4%
.12203992	86.6%
.14498943	97.0%
.13368309	92.1%

Subject Variables
ID

ced by prior

Sig.	95% Confidence Interval	
	Lower Bound	Upper Bound
.000	.000468	.000827
.000	.003478	.008459

ence Interval
Upper Bound
.061
.160
.329

ence Interval
Upper Bound
.173
.180

95% Confidence Interval	
Lower Bound	Upper Bound
-.036	.071
-.024	.083
.075	.182
.062	.170

.226	.333
.250	.357
